# Effects of Simulated Space Radiations on the Tomato Root Proteome

**DOI:** 10.3389/fpls.2019.01334

**Published:** 2019-10-24

**Authors:** Angiola Desiderio, Anna Maria Salzano, Andrea Scaloni, Silvia Massa, Maria Pimpinella, Vanessa De Coste, Claudio Pioli, Luca Nardi, Eugenio Benvenuto, Maria Elena Villani

**Affiliations:** ^1^Division Biotechnologies and Agroindustry, National Agency for Energy, New Technologies and Sustainable Economic Development (ENEA), Rome, Italy; ^2^Proteomics and Mass Spectrometry Laboratory, ISPAAM-National Research Council, Naples, Italy; ^3^National Institute of Ionizing Radiation Metrology, ENEA-INMRI, Rome, Italy; ^4^Division Health Protection Technologies, ENEA, Rome, Italy

**Keywords:** tomato hairy roots, ionizing radiations, Bioregenerative Life Support System, anthocyanins, stress response

## Abstract

Plant cultivation on spacecraft or planetary outposts is a promising and actual perspective both for food and bioactive molecules production. To this aim, plant response to ionizing radiations, as an important component of space radiation, must be assessed through on-ground experiments due to the potentially fatal effects on living systems. Hereby, we investigated the effects of X-rays and γ-rays exposure on tomato “hairy root” cultures (HRCs), which represent a solid platform for the production of pharmaceutically relevant molecules, including metabolites and recombinant proteins. In a space application perspective, we used an HRC system previously fortified through the accumulation of anthocyanins, which are known for their anti-oxidant properties. Roots were independently exposed to different photon radiations, namely X-rays (250 kV) and γ-rays (Co^60^, 1.25 MeV), both at the absorbed dose levels of 0.5, 5, and 10 Gy. Molecular changes induced in the proteome of HRCs were investigated by a comparative approach based on two-dimensional difference in-gel electrophoresis (2D-DIGE) technology, which allowed to highlight dynamic processes activated by these environmental stresses. Results revealed a comparable response to both photon treatments. In particular, the presence of differentially represented proteins were observed only when roots were exposed to 5 or 10 Gy of X-rays or γ-rays, while no variations were appreciated at 0.5 Gy of both radiations, when compared with unexposed control. Differentially represented proteins were identified by mass spectrometry procedures and their functional interactions were analyzed, revealing variations in the activation of stress response integrated mechanisms as well as in carbon/energy and protein metabolism. Specific results from above-mentioned procedures were validated by immunoblotting. Finally, a morphometric analysis verified the absence of significant alterations in the development of HRCs, allowing to ascribe the observed variations of protein expression to processes of acclimation to ionizing radiations. Overall results contribute to a meaningful risk evaluation for biological systems exposed to extra-terrestrial environments, in the perspective of manned interplanetary missions planned for the near future.

## Introduction

Plant cultivation is a key requirement for the success of long-term space missions. In fact, higher plants represent an essential component of bioregenerative life support systems (BLSS) for *in situ* production of food and pharmaceutical active molecules, not dependent on the supply at the launch or on periodic provision from Earth. However, the perspective of plant growth in the extraterrestrial environment raises the problem of the biological response to extreme conditions, including ionizing radiations, which are known to deposit energy inside living tissues causing structural and functional damages.

Current knowledge on the response of plants to radiation is based mainly on studies conducted in areas affected by nuclear accidents (Møller and Mousseau, 2016). These studies highlight a high variability in response to radiation stress based on plant species and the role of hormesis (i.e., a dose/biological response relationship) in adaptation to radiation is not sufficiently supported by bibliographic data. However, it is known that plants are more resistant to radiation than animals, including humans (Caplin and Willey, 2018). Hypotheses have been made to explain plants relative tolerance, such as higher efficiency in repairing DNA double strand breaks (Yokota et al., 2005) or higher basal rates of DNA methylation (Pecinka and Mittelsten Scheid, 2012). This higher resistance could be the result of evolutionary adaptation, which allowed plants to colonize land surface when ionizing radiations in the primordial Earth’s atmosphere were significantly higher than at present (Gensel, 2008). However, physiological mechanisms that regulate this higher tolerance are not completely elucidated, particularly in the perspective of plant growth during space missions (Arena et al., 2014) or in terrestrial environments contaminated by radiations (Danchenko et al., 2009).

Since the most detrimental effects of ionizing radiations, such as X-rays and γ-rays, are linked to DNA damage, the ability of a living organism to respond to a radiative injury (i.e., repairing the damage or activating compensatory molecular mechanisms) goes through the modulation of protein expression. In fact, proteins can directly repair the genome, regulate the accumulation of reactive oxygen species (ROS), or eliminate damaged macromolecules (Krisko and Radman, 2013). Therefore, dedicated studies based on proteomic analysis of plants exposed to ionizing radiations should provide comprehensive and explicative information about plant response to stress conditions and eventual acclimation mechanisms to counteract alterations.

The effect of radiation depends on different factors, which include type of radiation, biological characteristics of the irradiated tissue/organ/organism, dose, exposure and recovery time, and synergistic effect with other possible stressful agents (De Micco et al., 2011). Electromagnetic ionizing radiations, X-rays and γ-rays, which mainly differ for energetic characteristics, are the most dangerous due to their high penetration power into the matter, including protective barriers (Reisz et al., 2014). The probability of biological damage depends not only on the absorbed dose but also on the radiation ability to transfer energy through molecular collisions. This last property is measured as linear energy transfer (LET), which has relatively low values for X- and γ-rays. This means that, for the same dose, the direct damage caused on the biological molecules by these radiations is lower than that induced by high LET radiations (alpha particles, neutrons and protons). Despite the similar nature of X- and γ-rays, the biological effects caused for the same dose and exposure time may not be totally equivalent, due to the relative higher penetration power of γ-rays than X-rays (Hunter and Muirhead, 2009).

Moreover, the ionizations produced by X- and γ-rays have significant indirect effects, generating potent intracellular oxidants (H_2_O_2_, O_2_^•−^, and ^•^OH), along with reductants (H^•^ and e_aq_^−^), which in turn affect the living material as they yield energy (Reisz et al., 2014). This phenomenon has an impact on biological macromolecules, causing DNA breaks, protein fragmentation, or secondary–tertiary structure molecular deterioration, as well as cell membrane functional alteration with an increase of corresponding permeability. Hence, the capacity of the biological system to survive irradiation depends both on the extent of the damage and on its ability to repair the suffered molecular injury (Thompson, 2012).

It has been estimated that crews aboard the International Space Station (ISS) receive a total average dose of 80–160 mSv in a 6-month stay, depending on variations in the solar magnetic field that deflects the ionizing particles. A 3-year Mars mission could reach an overall equivalent dose of about 1 Sv, assuming the absence of frequent and intense solar particle events (SPEs) (Hellweg and Baumstark-Khan, 2007). Although these doses might not be lethal for a plant system (Caplin and Willey, 2018), especially if associated with a low dose rate, little is still known about the mechanisms that plants elicit to counteract these radiative stresses.

In this work, we evaluated the effects of both X- and γ-rays on “hairy root” cultures (HRCs), a recognized plant expression platform for the production of valuable molecules, offering advantages, e.g., containment, established cultivation conditions in hormone-free media, and product homogeneity, particularly in the case of industrial-scale production of secondary metabolites (Mirapleix et al., 2013). We used HRC from tomato (cultivar MicroTom) optimized for the expression of high levels of anthocyanins (Villani et al., 2017), which are endowed with anti-oxidizing properties thus offering more change to counteract the effect of cosmic radiations. For this plant system, we chose exposure doses ranging from 500 mGy up to 10 Gy (for proteomic analysis) and 20 Gy (for morphometric analysis), which are doses lethal for humans (Donnelly et al., 2010). These highest doses were considered as a simulation of an extreme stress that could occur during accidental exposure to high radiations. In the above-mentioned contexts, these experiments were performed with the aim to foresee the ability of plants to withstand high doses of radiation and to characterize the possible molecular effects caused by corresponding radiation stresses.

## Materials and Methods

### Tomato Hairy Roots Model

*In vitro* culture of tomato roots accumulating anthocyanins were obtained according to a procedure previously described (Villani et al., 2017). Briefly, tomato (cultivar MicroTom) leaf explants were infected with recombinant *Agrobacterium rhizogenes* (A4RSII strain, ATCC collection, Manassas VA 20108 USA) harboring a gene construct including a Myb-like transcription factor gene from *Petunia* (kindly given by Prof. R. Koes and F. Quattrocchio, University of Amsterdam) (Massa et al., 2016). Resulting hairy root clones (HRCs) showed the typical secondary branching and an intense purple pigmentation. One representative purple clonal root line was grown in polystyrene 85-mm-diameter Petri dishes (Phoenix Biomedical) onto Murashige–Skoog selective solid medium supplemented with 3% sucrose and 25 mg/l kanamycin (MS3k), and maintained at 22°C. Root explants were propagated on fresh medium one week before exposure to radiations to allow their acclimation.

### Tomato Roots Exposure to γ-Rays

For γ irradiation of HRCs, a teletherapy-type Co-60 unit, AECL model Eldorado 6, available at the ENEA-INMRI was used. At the sample position (i.e., at 135 cm source distance), the radiation beam originated by the decay of the Cobalt-60 included 14% of scattered photons and had an average energy of 1.12 MeV. The dose rate was 0.12 Gy/min and the beam uniformity over the HRC samples within 0.5%.

Three absorbed dose levels were delivered: 0.5 Gy (250 s irradiation time), 5 Gy (2,500 s irradiation time), and 10 Gy (5,000 s irradiation time). HRCs grown on agar medium in Petri dishes (diameter 7 cm) were fixed perpendicular to the beam axis, irradiating three independent biological replicates at each established dose, necessary to give statistical consistency to the proteomic analysis. In addition, three HRC samples were irradiated with an absorbed dose of 20 Gy (10,000 s irradiation time) for the morphometric analysis. As an experimental control, three non-irradiated HRC plates were treated in the same way as the exposed HRC plates (held for the same time in the same environment but away from γ-rays).

Once the irradiation was complete, the root cultures were moved into a growth chamber at a constant temperature of 22°C for 3 days, in order to allow the physiological recovery and the activation of response mechanisms induced by the stress imparted. After this time, the roots were frozen in liquid nitrogen and then stored at −80°C before proteomic analysis.

### Tomato Roots Exposure to X-Rays

X-ray exposure was achieved using a CHF 320G X-ray generator, operating at 250 kV and 15 mA, using 2.0 mm Al and 0.5 mm Cu filters. HRCs were exposed to the same doses adopted for γ radiation (0.5, 5, and 10 Gy), at a dose rate of 0.96 Gy/min. Three biological replicates were irradiated under each exposure condition; three non-irradiated cultures were used as control. As for γ exposure, after radiation, HRCs were moved to the growth chamber for 3 days. Then the roots were frozen in liquid nitrogen and stored at −80°C before proteomic analysis.

### Protein Extraction and Purification

Total protein extraction was optimized for anthocyanin-rich hairy roots, consisting in trichloroacetic acid (TCA)-acetone precipitation (Di Carli et al., 2011), which was combined with a second precipitation in ammonium acetate to remove the residual pigments. Briefly, hairy roots tissues (between 0.6 and 1.5 g) were ground in a mortar under liquid nitrogen. The resulting powders were finely homogenized using an Ultraturrax homogenizer (IKA) in 4 vol of 10% w/v TCA, 2% w/v dithiothreitol (DTT) in cold acetone containing protease inhibitor cocktail Complete (Roche); then the proteins were precipitated at −20°C, overnight. The pellets obtained after centrifugation at 8,000 *g* for 1 h, at 4°C, were washed with 0.07% w/v DTT in cold acetone, and incubated at −20°C, for 1 h. Proteins were collected by centrifugation at 8,000 *g*, for 1 h, at 4°C, and further washed at least three times with cold acetone (until the supernatant was colorless). Protein pellets were dried at room temperature and resuspended in lysis buffer (10 mM Tris-HCl, pH 8.0, 5 mM magnesium acetate, 8 M urea, 2% w/v ASB-14). Protein solutions were then subjected to a new precipitation in 5 vol of 0.1 M ammonium acetate in cold methanol, followed by washing in cold 80% v/v acetone. Again protein pellets were dried at room temperature and resuspended in lysis buffer. The protein extracts were purified using Clean Up kit (GE Healthcare) and quantified using the DC Protein Assay (BioRad), according to the manufacturer’s instructions. Proteins were then analyzed by one-dimensional gel electrophoresis (12% SDS-PAGE) and gels were silver-stained according to Oakley et al. (1980).

### 2D-DIGE

For the proteomic analysis of exposed HRCs, 2D-DIGE technology (two-dimensional difference in-gel electrophoresis, GE Healthcare) was used. 2D-DIGE technology has been specifically developed to drastically reduce experimental variability, allowing a reliable and statistically rigorous analysis (Diez et al., 2010). For this purpose, all the samples were analyzed simultaneously. On each electrophoresis gel, two distinct protein samples, labeled with different fluorophores (CyDyes DIGE Fluors: Cy3 and Cy5), were loaded. Moreover, an internal standard, consisting of a pool of all the samples under analysis, was labeled with a third fluorophore (Cy2) and was run on all gels, according to experimental design showed in **Table 1**. This allows to normalize signals from different gels, making spot matching and quantitation much simpler and statistically accurate. For protein labelling, each dye was diluted with anhydrous dimethylformamide and 50 µg of each protein extract was mixed with 200 pmol of amino-reactive cyanine dyes, and incubated for 30 min, in the dark. One microliter of 10 mM lysine was then added to quench the remaining free NHS esters of the cyanine dyes, incubating in the dark for 10 min. An equal volume of 2× sample buffer (7 M urea, 2 M thiourea, 130 mM DTT, 2% w/v ASB-14, 2% IPG buffer 4-7) was added, and the samples were incubated for 10 min, in the dark. IEF rehydration buffer (7 M urea, 2 M thiourea, 13 mM DTT, 2% w/v ASB-14, 1% IPG buffer 4–7) was added to each sample to obtain 350 µl final volume and the protein solution was used to passively rehydrate IPG-strips (pH 4–7/18 cm, GE Healthcare), overnight, at room temperature. Isoelectrofocusing (IEF) was performed on an IPGphor 3 unit (GE Healthcare) in order to obtain the first dimension separation of HRPs’ proteome. After IEF, each strip was incubated for 15 min in 20 ml equilibration buffer (50 mM Tris-HCl, pH 8.8, 6 M urea, 30% v/v glycerol, 2% w/v SDS, traces of bromophenol blue), containing 1% w/v DTT to reduce proteins. Proteins were then alkylated in the presence of 2.5% w/v iodacetamide in equilibration buffer, for 15 min, at room temperature. The second dimension separation was obtained using Ettan Dalt Twelve unit (GE Healthcare) and 10% polyacrylamide gels (18 cm × 20 cm × 1 mm) in running buffer (250 mM Tris-HCl, pH 8.3, 1.92 M glycine, 1% w/v SDS). The electrophoresis was performed at 15°C, applying 2 W/gel for 15 min and 20 W/gel for further 4–5 h. For 2D-DIGE analysis of HRCs exposed to γ-rays and X-rays, a total of seven gels were run, respectively, i.e., six analytical gels for the separation of the three biological replicates for each radiation dose (control, 0.5, 5, and 10 Gy), and one preparative gel for protein spot picking.

**Table 1:** Two-dimensional difference in-gel electrophoresis (2D-DIGE) experimental design.

Gel number	Cy2 internal standard	Cy3 sample	Cy5 sample
**1**	50 µg (4.17 µg each of samples)	50 µg sample C (1)	50 µg sample 0.5 Gy (1)
**2**	50 µg (4.17 µg each of samples)	50 µg sample 5 Gy (1)	50 µg sample C (2)
**3**	50 µg (4.17 µg each of samples)	50 µg sample C (3)	50 µg sample 10 Gy (1)
**4**	50 µg (4.17 µg each of samples)	50 µg sample 0.5 Gy (2)	50 µg sample 5 Gy (3)
**5**	50 µg (4.17 µg each of samples)	50 µg sample 5 Gy (2)	50 µg sample 10 Gy (3)
**6**	50 µg (4.17 µg each of samples)	50 µg sample 10 Gy (2)	50 µg sample 0.5 Gy(3)

Three biological replicates of protein samples (indicated by numbers in parenthesis) were analyzed for each condition (unexposed control C; 0.5, 5, and 10 Gy exposed). The same experimental design was adopted for “hairy root” cultures (HRCs) exposed both to γ- and X-rays.

### Proteomic Profile Analysis

Protein maps obtained after 2D electrophoretic separation were visualized with a Typhoon 9410 Imager (GE Healthcare) set at the appropriate wavelengths for each dye. The images were then exported to the Batch Processor of DeCyder 2D Software v 7.2 (GE Healthcare) and statistically elaborated by Biological Variation Analysis module, as already described (Di Carli et al., 2011). Univariate analysis one-way ANOVA was performed and protein spots with a statistically significant variation (*p* ≤ 0.05, fold change over 1.5, filtered for false discovery rate) were detected as differentially represented and automatically isolated from gel by the Ettan Spot Picker System (GE Healthcare). Multivariate analysis, consisting of hierarchical clustering analysis (HCA) and principal component analysis (PCA), was performed using the DeCyder-EDA (Extended Data Analysis) module.

### Protein Identification by Mass Spectrometry Analysis

Spots from 2D-DIGE were excised, reduced with DTT, alkylated with iodoacetamide, and digested with trypsin as previously reported (D’Ambrosio et al., 2006). Protein digests were subjected to a desalting step on μZipTipC18 pipette tips (Millipore) and then analyzed by nano-liquid chromatography (nLC)–electrospray ionization (ESI)–tandem mass spectrometry (MS/MS) using LTQ XL and Q Exactive Plus instruments (Thermo Fischer Scientific, USA), both equipped with UltiMate 3000 HPLC RSLC nano systems (Dionex, USA). Peptides were resolved on an Easy C18 column (100 × 0.075 mm, 3 μm), at a flow rate of 300 nl/min, using a gradient elution with a mixture of water/acetonitrile in 0.1% formic acid, as already reported (Salzano et al., 2013). When an LTQ XL instrument was used, full mass spectra were acquired in the range *m/z* 400–1,400, and data-dependent automatic MS/MS acquisition was applied to the three most abundant ions (Top3), enabling dynamic exclusion with repeat count 1 and exclusion duration 60 s. For MS/MS analysis mass isolation window and collision energy were set to *m/z* 3 and 35%, respectively. When a Q Exactive Plus instrument was used, mass spectrometry analysis was performed as recently described (Lonoce et al., 2019).

Raw data from nLC-ESI-MS/MS analysis were searched by MASCOT v2.6.2, (Matrix Science, UK) within Proteome Discoverer v2.1, against a *Solanum lycopersicum* protein sequence database retrieved from UniProtKB repository (39,642 sequences, 01/2017). The following parameters were used for protein identification: a mass tolerance value of 2 Da for precursor ion and 0.8 Da for MS/MS fragments, trypsin as proteolytic enzyme, a missed-cleavages maximum value of 2, Cys carbamidomethylation as fixed modification, Met oxidation, and Gln- > PyroGlu formation as variable modifications. Protein candidates with at least two significantly matched peptide sequences (expectation value < 0.05) with ion score > 30 were further evaluated by comparison of their experimental molecular mass and pI values with their theoretical counterparts. Definitive protein assignment was always associated with manual spectra visualization and verification. Identified proteins were further filtered according to an EMPAI ratio criterion (EMPAI 1st/EMPAI 2st > 2), which is conventionally used to exclude minor proteins not contributing to quantitative changes detected by 2D-DIGE (Shinoda et al., 2010), and finally subjected to BLAST analysis against the TAIR 10 protein sequence database from The Arabidopsis Information Resource (TAIR) repository (www.arabidopsis.org). Proteomic data have been deposited to the ProteomeXchange Consortium (Vizcaíno et al., 2016) *via* the PRIDE partner repository with the dataset identifier PXD014748.

### Analysis of Protein–Protein Interactions

Proteins identified as differentially represented after exposure to ionizing radiation were imported into the online Search Tool for the Retrieval of Interacting Genes/Proteins (STRING) database v11.0 (http://string-db.org; Szklarczyk et al., 2015) for known and predicted protein–protein interactions (PPIs). In order to minimize the rate of false positives, PPIs confirmed by experimental study, pathways from curated databases and reported in abstracts of papers published in PubMed were selected. The interactions comprised both direct (physical) and indirect (functional) associations between proteins.

### Immunoblot Analysis

Immunoblot analysis was used to validate differential expression data obtained by 2D-DIGE analysis. To this aim, we selected two proteins differentially represented both after γ-rays and X-rays exposure, namely, enolase and chloroplastic ATP synthase subunit B. As a standard for normalization of protein quantity, actin was used. The same samples extracted for 2D-DIGE were analyzed. HRC purified extracts (1.5 μg total soluble proteins) were solved in 80 mM Tris-HCl, pH 6.8, 10% v/v glycerol, 2% v/v SDS, 143 mM β-mercaptoethanol, and traces of bromophenol blue, and heated for 5 min, at 100°C. Proteins were then separated by 12% SDS-PAGE and electrotransferred to polyvinylidene difluoride (PVDF) membrane. After blotting, membranes were blocked in 5% milk in phosphate buffer saline (PBS, 137 mM NaCl, 2.7 mM KCl, 10 mM Na2HPO4, 1.8 mM KH2PO4), ovenight. Membranes were washed three times in PBS containing 0.5% w/v Tween, for 10 min per wash, and then incubated with primary specific antibodies obtained from Agrisera (Sweden), for 1.5 h, at room temperature. All antibodies were diluted in 2.5% milk in PBS. Actin was revealed with anti-actin rabbit polyclonal antibody diluted 1:5,000; chloroplastic ATP synthase subunit B was revealed with anti-AtpB rabbit polyclonal antibody diluted 1:5,000; enolase was revealed with anti-enolase rabbit polyclonal antibody diluted 1:2,000. The anti-actin antibody was used in coincubation with anti-AtpB or anti-enolase. Membranes were then washed three times with PBS containing 0.5% w/v Tween, and incubated 1.5 h, at room temperature, with an anti-rabbit secondary antibody conjugated with horseradish peroxidase (Sigma), which was diluted 1:5,000 in 2.5% milk in PBS. After washing as described above, chemiluminescence was revealed using ECL Prime (GE Healthcare) following the manufacturer’s instructions. Protein representation was measured by a densitometric analysis using ImageQuant TL 1D v8.1 software. Enolase and chloroplastic ATP synthase subunit in HRC extracts were quantified as the ratio to the amount of actin.

The statistical significance of differences in protein relative abundance obtained by immunoblotting was calculated by one-way ANOVA, followed by Fisher’s least significant difference (LSD) test, by using Prism 8 Software version 8.1.1. Each group was composed by three biological replicates. Separate analyses for enolase and AtpB were performed on samples exposed to γ-rays and X-rays.

### Morphometric Analysis

The effects on HRC growth of γ radiation exposure at a dose of 20 Gy (dose rate of 0.12 Gy/min) were analyzed on three biological replicates of roots of approximate length 5 cm, propagated on fresh MS3k solid medium in Petri dishes and kept in growth chamber at 22°C. HRC images were obtained by scanning, using CanoScan LiDE 25 (Canon), and analyzed by the biometric software EZ-Rhizo (Armengaud et al., 2009), as already described (Villani et al., 2017).

## Results and Discussion

Plant colonization of land surface started around 460 million years ago, when the levels of β/γ-radiation in the Earth’s atmosphere were significantly higher than at present (Caplin and Willey, 2018). These environmental stresses have driven the evolution of terrestrial plant organisms, imposing the development of mechanisms of adaptation to radiations, which in all probability are partly maintained even in modern species. The association between the larger effects of radiation for plants than animals and the sedentary nature of the former, which are unable to move away from exposed areas, should result in a greater level of their local adaptation (Møller and Mousseau, 2013). Moreover, exceptional and disastrous events, such as the explosion of the Chernobyl nuclear power plant, highlighted the extraordinary ability of plants, e.g., soybean, to survive and adapt to an environment with a quantity of radioactive isotopes up to 163-fold higher than normal (Danchenko et al., 2009).

These assumptions support the prospect of using plants for the formulation of bioregenerative systems for life support in space (BLSS). Nevertheless, it is necessary to know in depth the response of plant systems by simulating the conditions that could actually occur during space missions. The aim of this work is to investigate about the limits of the ability to acclimatize and the molecular mechanisms of adaptation to extreme conditions. This last aspect is particularly important in the need to prevent possible negative effects on development/maturation and on the accumulation of undesired components in the plant product destined for human consumption.

In this study, we chose a tomato dwarf variety (MicroTom cultivar) as a model system; this cultivar has already been proposed for cultivation in space environment based on its favorable characteristics both from the cultural and nutritional point of view (Arena et al., 2019). Created for ornamental purposes, this variety has been widely used as a model plant in other characterization studies. The small size (average height 15 cm), the short life cycle (fruit production in 3 months), the high productivity independent from the photoperiod, and the autogamous pollination (Martí et al., 2006) are among the characteristics that make this variety an excellent candidate for cultivation during space missions for the production of fresh food. Furthermore, other authors already demonstrated the ability of this variety to cope with radiation stress through adjustments at the physiological level (Arena et al., 2019).

Among the known effects of ionizing radiation, there is the activation of processes that determine the accumulation of ROS, capable of inducing cell damage. Plant survival after exposure relies on the ability to counteract oxidative stress through the production of compounds with antioxidant properties (i.e., anthocyanins and vitamin C) (Dixit et al., 2010; Gill and Tuteja, 2010), and the activation of anti-oxidant enzymes (Esnault et al., 2010; De Micco et al., 2011). This consideration has guided us in identifying a suitable plant “ideotype” (i.e., a genotype with the best characteristics for the specific environment) as a plant system to study the effects of space radiations. In this work, we focused on a system of MicroTom roots in culture (hairy roots), aimed at producing ready-to-use biopharmaceuticals. With the aim of optimizing the performances of this plant ideotype in the space environment, we used MicroTom hairy roots bioengineered to promote the accumulation of anthocyanins, which are pigments known for their antioxidant properties. Although there are very few papers published on the response of roots to radiation stresses, it is well known that the effect of irradiation, also appreciated as a variation of gene expression, is more pronounced in roots than in shoots (Biermans et al., 2015).

### Proteome Response to Ionizing Radiations

In order to analyze tomato HRC system response to the on-ground simulation of radiative stress, three experimental conditions were identified for both X and γ irradiation. As a minimum dose, the roots were exposed to 0.5 Gy to mimic conditions that could actually occur during long-term missions (i.e., expected total dose absorbed during a mission to Mars or during SPEs; Wu et al., 2009). A higher dose of 10 Gy was chosen to evaluate the response of the root in extreme, but not lethal, conditions of radiation (i.e., exposure to exceptional events, such as a solar storm). Moreover, an intermediate dose of 5 Gy was added to this experimental design to verify the activation of specific dose-dependent responses to above-mentioned stresses.

Since the direct radiation damage due essentially to structural alterations of macromolecules is immediate, we waited three recovery days before performing proteomic analysis with the aim to better appreciate the response of HRCs to irradiation with both X- and γ-rays. The rationale for this choice was based on the consideration that radiation triggers a cascade of molecular processes, also involving oxidizing effects caused by ROS production, whose full manifestation takes a few days (Gudkov et al., 2019). During this period, radiation-induced dysfunctions of many processes become more evident and/or damage compensation mechanisms are put in place, making the analysis more informative.

**Figure 1:**
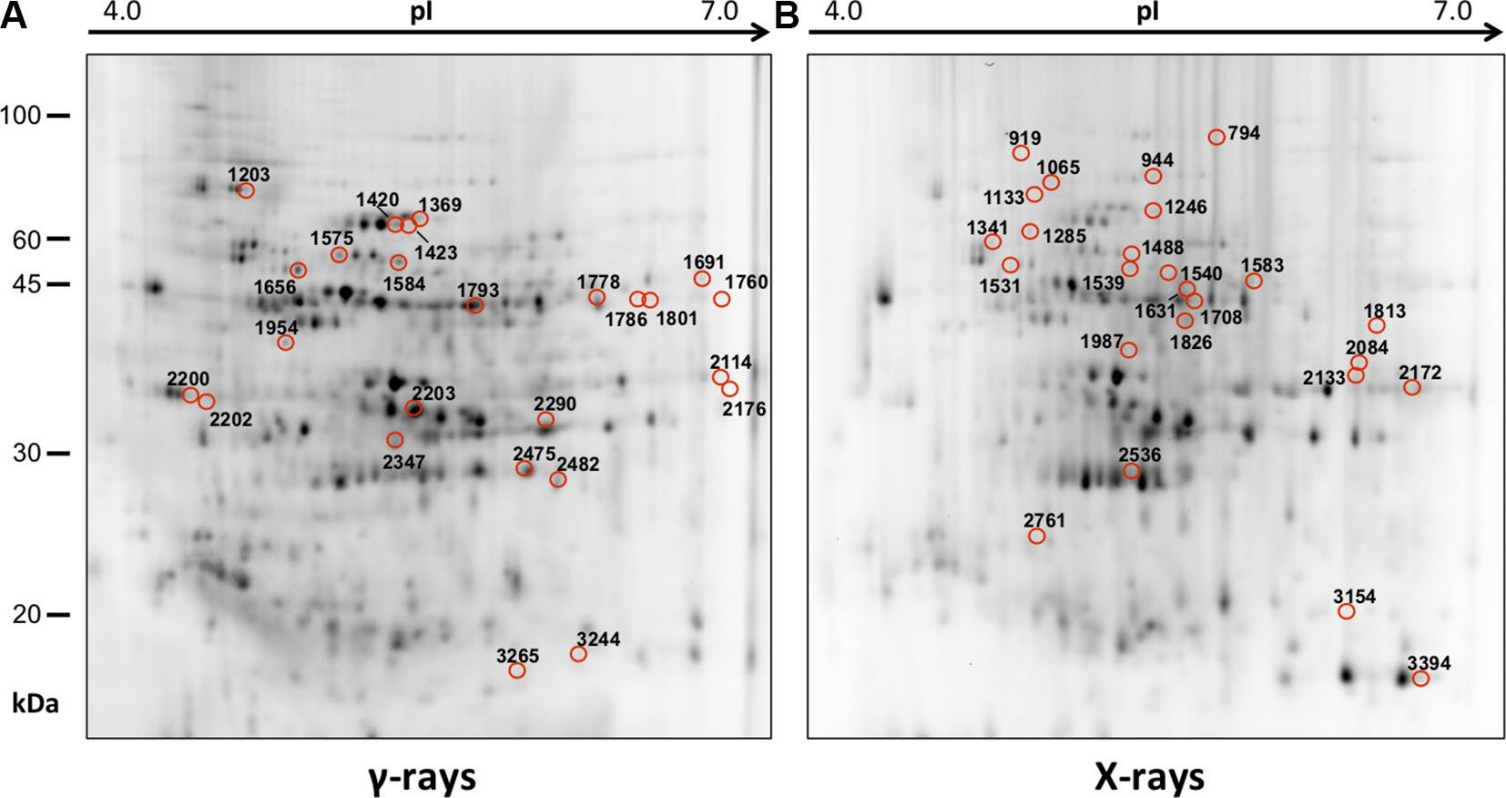
Protein maps obtained from two-dimensional difference in-gel electrophoresis (2D-DIGE) analysis of “hairy root” culture (HRC) exposed to γ-rays **(A)** and X-rays **(B)**. Silver-stained 2D gels are represented. Differentially represented protein spots (DRPs), indicated by circles, were isolated from the gel and analyzed by mass spectrometry. The corresponding list is reported in **Tables 1** and **2**.

In order to obtain an adequate representativeness of the soluble proteins, an extraction protocol was specifically developed for the plant material under examination, which allowed to obtain 0.2 mg of purified total proteins from 1 g of fresh HRC tissues. For the subsequent proteomic analysis of these extracts, the 2D-DIGE technology was used. Three biological replicates of the hairy roots fortified through the accumulation of anthocyanins were analyzed for each exposure condition, in order to minimize the experimental variability, applying a reliable and statistically rigorous comparative analysis. Protein samples obtained from HRCs independently exposed at different doses of X-rays and γ-rays were analyzed in comparison to unexposed HRCs (complete set of protein maps in **Supplementary Figures S1** and **S2**). An average of 1,005 (coefficient of variation 15%) protein spots were resolved (MW range: 10–200 kDa; pI range: 4–7) for γ-rays response analysis, and 983 (coefficient of variation 18%) for X-rays exposure. Samples exposed to radiation at 0.5 Gy showed no statistically significant variation of protein representation profiles (i.e., no differentially represented protein spots—DRPs—obtained from one-way ANOVA even for *p* value < 0.1) for both data sets obtained after irradiation with X-rays and γ-rays. Conversely, an alteration of protein representation levels was appreciated at 5 and 10 Gy. At these exposure conditions, statistical analysis of DRPs compared to the untreated counterpart identified 28 protein spots for γ-ray exposure analysis and 30 protein spots for X-ray exposure (**Figure 1**).

**Figure 2:**
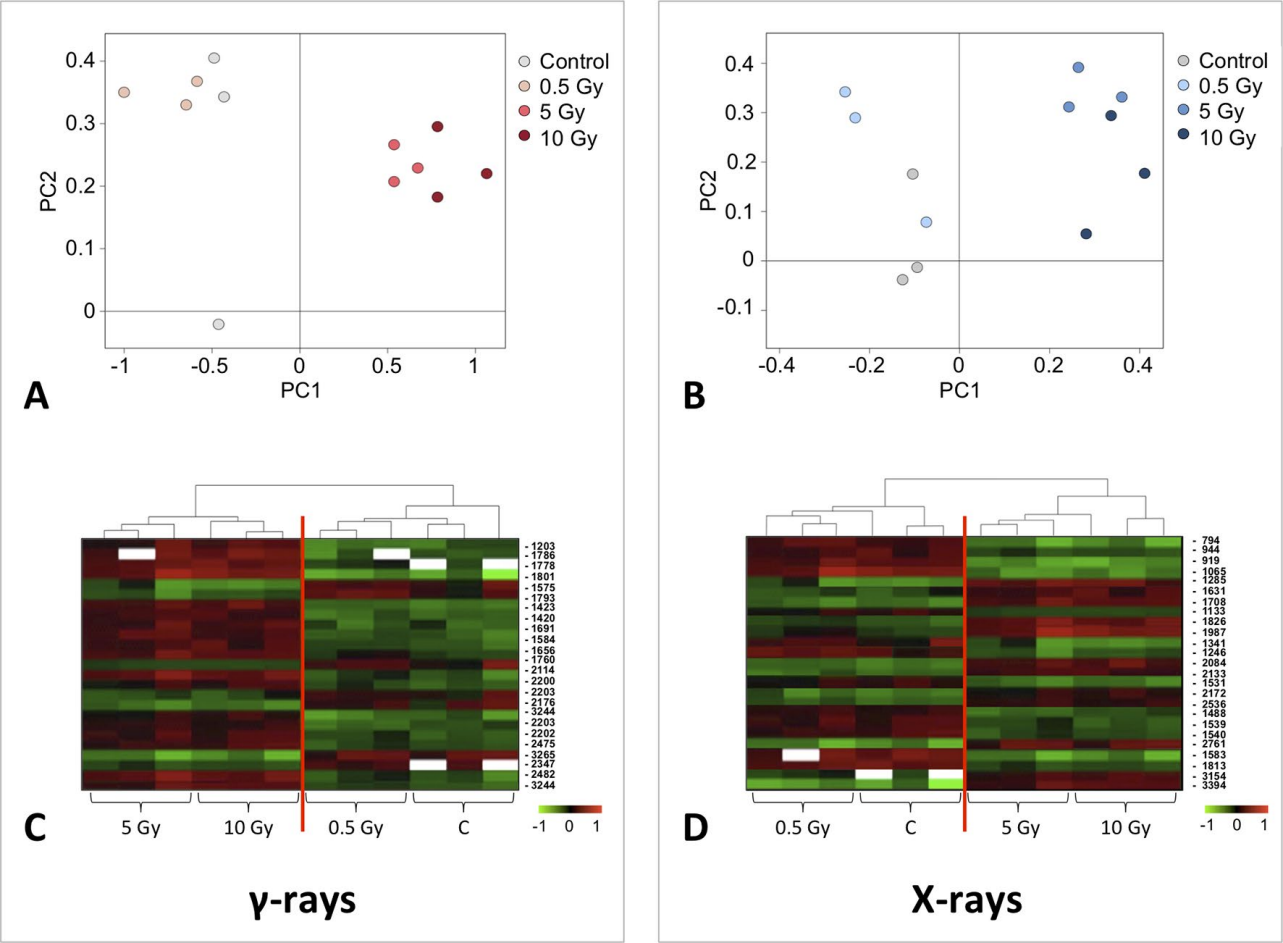
Principal component analysis (PCA) of differential represented spots (*p ≤* 0.05) obtained from the analysis of γ-rays **(A)** and X-rays **(B)** response. Each circle represents a spot map corresponding to a single HRC. Three HRCs (i.e., biological replicates) were independently exposed and analyzed for each irradiation condition, here displayed in different colors. The first two principal components PC1 and PC2 showed a variance of 78.1% and 11.7% for γ-ray analysis, and of 65.0% and 19.8% for X-ray analysis, respectively. Pattern analysis by hierarchical clustering (HCA) of the 25 differential spots for γ-rays response **(C)** and 25 differential spots for X-rays response **(D)** (listed on the right), based on their representation in the spot maps. The dendogram on the top of hierarchical clustering analysis (HCA) ordered the data so that similar data were displayed next to each other. HRC samples with similar expression profiles (i.e., similar expression over the spot maps) were clustered together. Red and green indicate overrepresented and downrepresented proteins according to the scale at the bottom of the figure.

A multivariate ANOVA was conducted in order to statistically discriminate between biological variability of biological replicates treated under the same experimental conditions and variability attributable to the physiological response to ionizing radiation. PCA was performed on 80% and 75% of the complete data set, for X-ray and γ-ray exposure, respectively (**Figures 2A, B**), with the aim to identify protein groups responsible for correlated variations. The first two principal components showed a variance of 78.1% and 11.7% for γ-ray analysis, and of 65.0% and 19.8% for X-ray analysis, respectively. PCA resulted in similar groupings between the elaborations of proteomic variations after exposure to γ-rays and X-rays. In fact, a clear separation was evident in the protein loading plots between two groups of samples, responding differently in terms of protein representation. A first grouping associated the roots exposed at 0.5 Gy and the untreated control, while a second grouping included the roots exposed to 5 and 10 Gy. This dose-dependent response to radiation was also confirmed by the hierarchical cluster analysis (HCA), so that the variations (over- or down-representation) after irradiation were evident only from 5 Gy, and remained approximately constant at 10 Gy (**Figures 2C, D**). This experimental evidence indicated that the HRC system under study tolerates radiation levels up to 0.5 Gy. At 5 Gy, metabolic processes are activated to counteract the effects of radiations, and these bioprotective mechanisms do not appear to be significantly affected by the radiation dose, at least up to 10 Gy.

**Figure 3:**
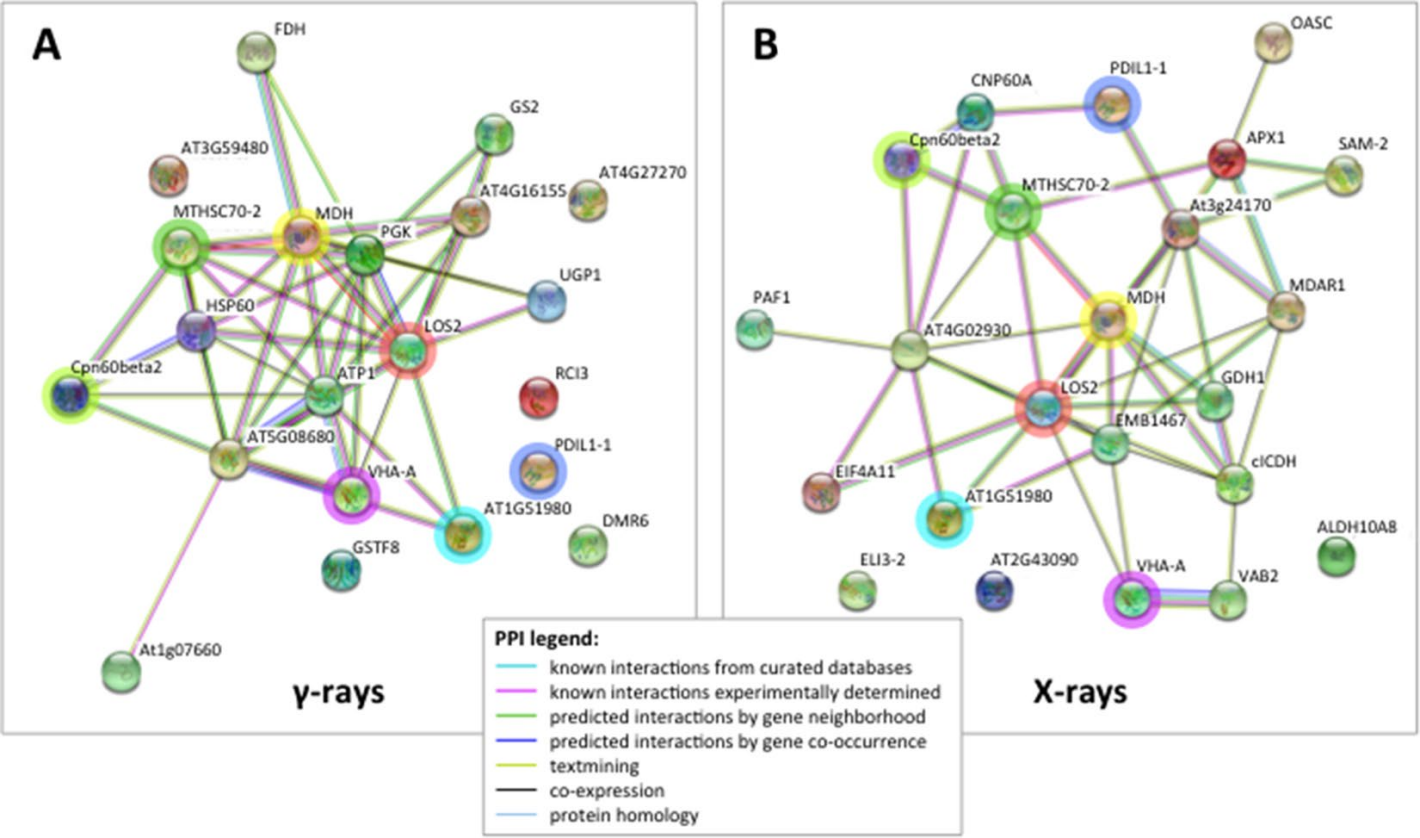
STRING protein–protein interaction (PPI) analyses. **(A)** PPI network connectivity for proteins identified as differentially represented after γ-ray exposure. The network contains 21 nodes with 45 edges (vs. 7 expected edges); clustering coefficient 0.579; enrichment *p*-value 1.0e-16; average node degree 4.3. **(B)** PPI network connectivity for proteins identified as differentially represented after X-ray exposure. The network contains 23 nodes with 46 edges (vs. 10 expected edges); clustering coefficient 0.481; enrichment *p*-value 2.2e-16; average node degree 4.0. PPI legends indicate the type of interaction evidence. The colored rings highlight seven identical proteins identified as differentially represented in both X and γ analysis, namely TCP-1/cpn60 Chaperonin (Cpn60beta2), mitochondrial Heat Shock Protein 70 (MTHSC70-2), malate dehydrogenase (MDH), enolase (LOS2), vacuolar ATP synthase subunit A (VHA-A), protein disulfide isomerase (PDIL1-1), and insulinase (AT1G51980).

Twenty-five protein spots obtained in response to 5–10 Gy of γ-rays and X-rays (**Tables 2** and **3**, respectively) were identified by mass spectrometric analysis, which allowed to characterize 23 and 21 variably represented proteins for X- and γ-ray analysis, respectively. For some spots (12 and 15 in number for X- and γ-ray analysis, respectively), identification was straightforward since a unique component was ascertained therein. In other cases, two to three proteins co-migrated within the same spot (**Supplementary Tables S1** and **S3**). After exclusion of non-influent proteins according to the EMPAI criterion reported in the experimental section (Shinoda et al., 2010), definitive component assignment to spot variations was done only after the recognition of a coherent quantitative trend of the same species, when it also occurred in other spots. On the other hand, seven common DRPs were found in HRCs subjected to both ionizing radiations. This indicated the occurrence of a significant overlap of the mechanisms activated in HRCs in response to the two radiation stresses (percentage of DRPs in common compared to the total DRPs identified: 33.3% for γ-ray and 30.4% for X-ray response analysis) (**Figure 3**).

**Table 2:** Protein spots recognized as differentially represented after γ-ray exposure and identified by mass spectrometry analysis.

Spot number	Protein identification	Accession number	Abbreviation (STRING reference)	Functional category	Relative protein abundance^(1)^	Average ratio^(1)^ (0.5 Gy; 5 Gy; 10 Gy/not exposed)	pI^(2)^ (Theor/Exp)	MW^(3)^ (kDa–Theor/Exp)	Coverage/N. peptides^(4)^
1203 1778	Enolase	AT2G36530.1	LOS2	Carbon metabolism	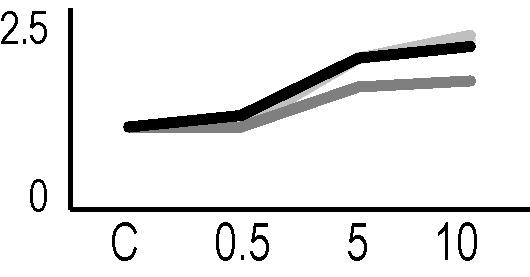	1203: 1.0; 1.9; 2.1 1778: 1.0; 1.9; 2.2 1786: 1.0; 1.5; 1.6 1801: 1.2; 1.9; 2.0	1203: 5.68/5.15 1778: 5.68/5.91	1203: 48.054/72.119 1778: 48 054/44.802	1203: 23.6/7 1778: 51.4/23
1203 1778 1786 1801	UDP-glucose pyrophosphorylase	AT3G03250.1	UGP1	Carbon metabolism			1203: 5.84/5.15 1778: 5.84/5.91 1786: 5.84/6.02 1801: 5.84/6.13	1203: 52.014/72.119 1778: 52.014/44.802 1786: 52.014/44.599 1801: 52.014/44.206	1203: 15.1/5 1778: 43.8/22 1786: 22.6/9 1801: 41.1/18
1369	Heat shock protein 70, mitochondrial	AT5G09590.1	MTHSC70-2	Protein folding/refolding	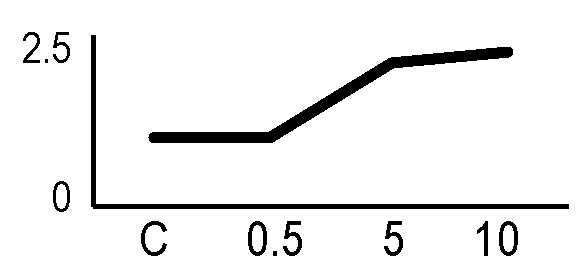	1369: 1.0; 2.1; 2.3	1369: 5.75/5.52	1369: 73.153/65.870	1369: 27.0/17
1420 1423	ATP synthase subunit A, vacuol	AT1G78900.2	VHA-A	Amino acid metabolism	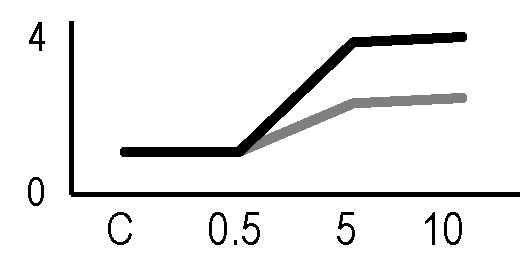	1420: 1.0; 2.1; 2.2 1423: 1.0; 3.5; 3.6	1420: 5.20/5.42 1423: 5.20/5.51	1420: 68.798/64.219 1423: 68.798/64.023	1420: 46.2/29 1423: 32.6/18
1575	Heat shock protein 60	AT3G23990.1	HSP60	Protein folding/refolding	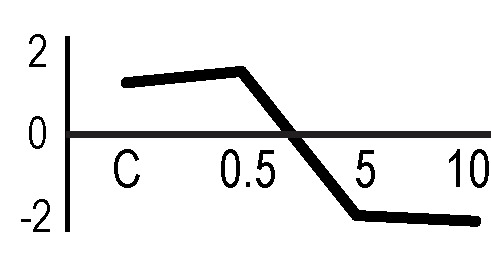	1575: 1.3; -1.7; -1.8	1575: 5.80/5.35	1575: 61.438/58.531	1575: 45.5/25
1584	TCP-1/cpn60 chaperonin family protein	AT3G13470.1	Cpn60beta2	Protein folding/refolding	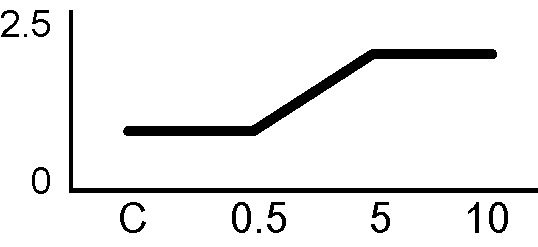	1584: 1.0; 2.3; 2.3	1584: 5.72/5.48	1584: 63.238/51.622	1584: 40.6/24
1656	Protein disulfide isomerase	AT1G21750.2	PDIL1-1	Protein folding/refolding	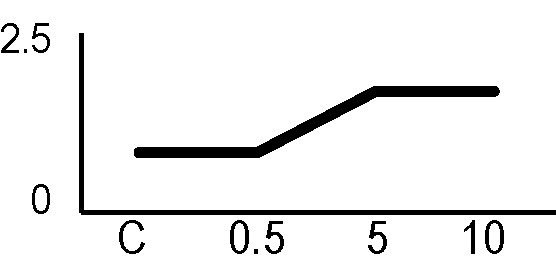	1656: 1.0; 2.0; 2.1	1656: 4.96/5.22	1656: 49.612/48.553	1656: 42.2/15
1691	ATP synthase subunit 1	ATMG01190.1	ATP1	Amino acid metabolism	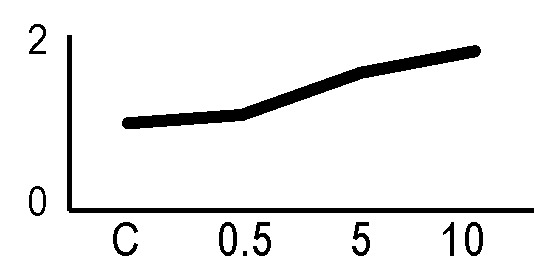	1691: 1.1; 1.6; 1.8	1691: 5.30/6.52	1691: 18.381/46.021	1691: 48.3/8
1760	Dihydrolipoyl dehydrogenase	AT4G16155.1	AT4G16155	Oxidation/reduction	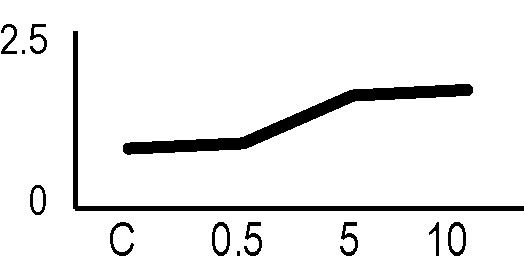	1760: 1.1; 1.9; 2.0	1760: 6.82/6.89	1760: 60.562/44.970	1760: 7.2/3
1793	Insulinase	AT1G51980.1	AT1G51980	Protein metabolism	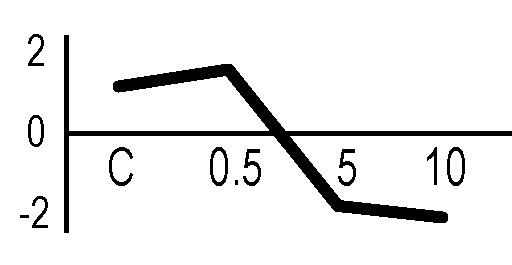	1793: 1.3; -1.5; -1.7	1793: 6.05/5.62	1793: 54.869/43.575	1793: 45.4/16
1954	Histone H4	AT5G59970.1	AT5G59970.1	Protein metabolism	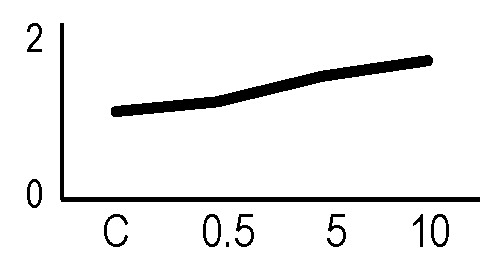	1954: 1.1; 1.4; 1.6	1954: 11.48/5.21	1954: 11.402/42.118	1954: 34.0/3
2114	Formate dehydrogenase	AT5G14780.1	FDH	Oxidation/reduction	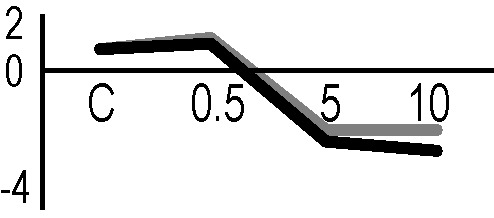	2114: 1.3; -1.6; -1.6 2176: 1.1; -2.0; -2.2	2114: 6.87/7.51	2114: 42.352/38.822	2114: 21.5/7
2114 2176	Peroxidase	AT1G05260.1	RCI3	Oxidation/reduction			2114: 8.03/7.51 2176: 8.03/7.82	2114: 36.427/38.822 2176: 36.427/36.455	2114: 17.4/5 2176: 20.1/6
2200 2203	ATP synthase subunit beta	AT5G08680.1	AT5G08680	Amino acid metabolism	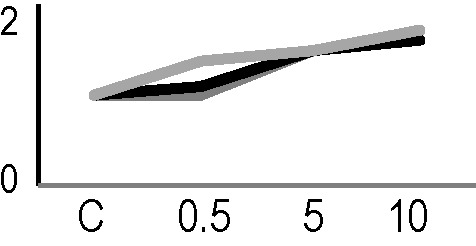	2200: 1.4; 1.5; 1.7 2203: 1.0; 1.5; 1.7 2290: 1.1; 1.5; 1.6	2200: 5.28/5.50 2203: 5.28/5.52	2200: 53.491/36.045 2203: 53.491/34.931	2200: 5.0/2 2203: 14.9/7
2203 2290	Glutamine synthetase	AT5G35630.3	GS2	Amino acid metabolism			2203: 6.29/5.52 2290: 6.29/5.80	2203: 47.852/34.931 2290: 47.852/33.572	2203: 13.0/7 2290: 16.9/11
2202	Phosphoglycerate kinase	AT1G79550.2	PGK	Protein folding/refolding	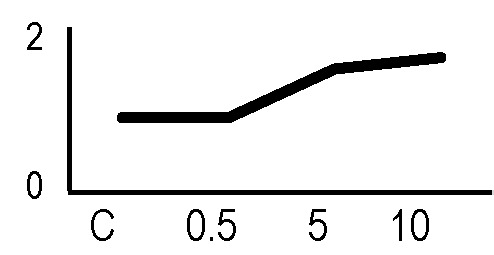	2202: 1.0; 1.6; 1.8	2202: 5.78/5.12	2202: 42.263/35.900	2202: 27.4/7
2347	Putative 2OG-Fe oxygenase	AT5G24530.1	DMR6	Oxidation/reduction	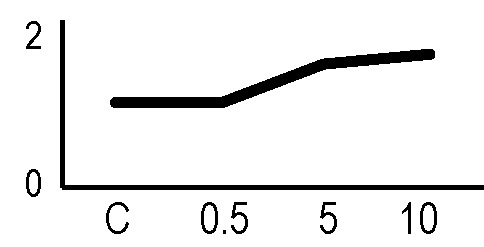	2347: 1.0; 1.5; 1.6	2347: 5.40/5.43	2347: 38.528/32.009	2347: 5.6/2
2475 2482	Fructokinase-2	AT3G59480.1	AT3G59480	Carbon metabolism	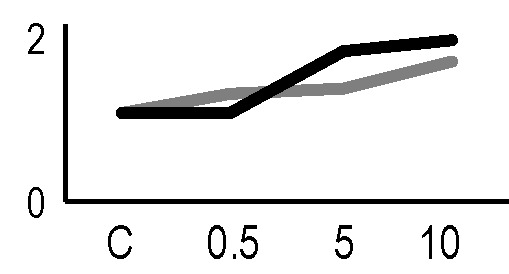	2475: 1.2; 1.3; 1.6 2482: 1.0; 1.7; 1.8	2475: 5.76/5.72 2482: 5.76/5.82	2475: 34.969/29.769 2482: 34.969/29.207	2475: 54.9/15 2482: 66.5/20
2475	Malate dehydrogenase	AT3G47520.1	MDH	Oxidation/reduction			2475: 8.34/5.72	2475: 43.563/29.769	2475: 34.0/13
3244 3265	Glutathione transferase	AT2G47730.1	GSTF8	Oxidation/reduction	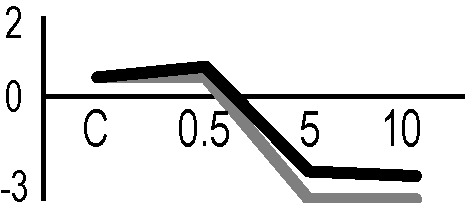	3244: 1.1 - 3.0 - 3.0 3265: 1.4 - 2.1 - 2.2	3244: 5.98/5.85 3265: 5.98/5.72	3244: 23.708/19.928 3265: 23.708/19.433	3244: 36.2/8 3265: 32.9/7
3265	Quinone reductase family protein	AT4G27270.1	AT4G27270	Oxidation/reduction			3265: 5.74/5.72	3265: 21.655/19.433	3265: 37.4/7

^(1)^Relative protein abundance represented as graphic display of the Average Ratio values obtained from the statistical analysis and calculated as the ratio between the representativeness values of the single spot in the samples exposed to 0.5, 5, or 10 Gy and those obtained in the unexposed samples. ^(2)^Experimental (Exp) and theoretical (Theor) isoelectric point (pI) values. ^(3)^Experimental (Exp) and theoretical (Theor) molecular weight (MW). ^(4)^Protein sequence coverage (%) and number of unique peptides identified for each spot. Identification details (including Mascot score) are reported in **Supplementary Table S1**, while results from sequence alignment with respect to Arabidopsis thaliana counterparts are reported in **Supplementary Table S2**.

**Table 3:** Protein spots recognized as differentially represented after X-ray exposure and identified by mass spectrometry analysis.

Spot number	Protein identification	Accession number	Abbreviation (STRING reference)	Functional category	Relative protein abundance^(1)^	Average ratio^(1)^ (0.5 Gy; 5 Gy; 10 Gy/not exposed)	pI^(2)^ (Theor/Exp)	MW^(2)^ (kDa–Theor/Exp)	Coverage/N. peptides^(3)^
794 944	NADH dehydrogenase, mitochondrial	AT5G37510.1	EMB1467	Oxidation/reduction	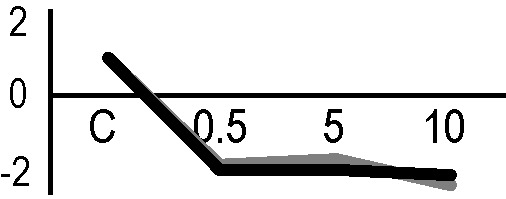	794: 1.3; -1.2; -1.8 944: 1.5; -1.5; -1.6	794: 5.90/5.90 944: 5.90/5.70	794: 80.768/85.071 944: 80.768/80.654	794: 37.9/17 944: 36.8/18
919 1133 1341 1065	Chaperonin 6 alpha	AT2G28000.1	CPN60A	Protein folding/refolding	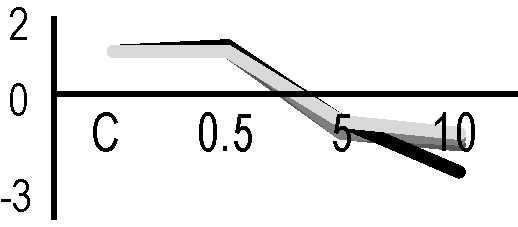	919: 1.0; -1.4; -1.8 1065: 1.2; -1.4; -1.7 1133: 1.1; -1.1; -2.6 1341: 1.0; -1.1; -1.5	919: 5.21/5.43 1133: 5.21/5.46 1341: 5.21/5.35 1065: 5.21/5.50	919: 62.032/82.563 1133: 62.032/73.201 1341: 62.032/58.881 1065: 62.032/78.322	919: 36.7/15 1065: 23.5/10 1133: 36.4/16 1341: 37.3/12
1246	Heat shock protein 70, mitochondrial	AT5G09590.1	MTHSC70-2	Protein folding/refolding	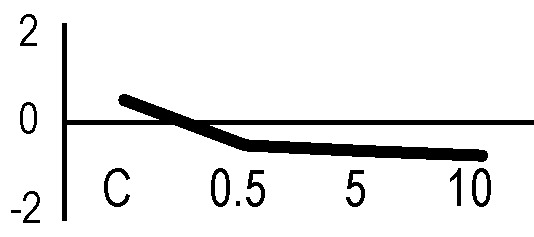	1246: -1.1; -1.4; -1.7	1246: 5.75/5.70	1246: 73.153/71.340	1246: 28.2/14
1246	ATP synthase, subunit A, vacuolar	AT1G78900.2	VHA-A	Amino acid metabolism			1246: 5.20/5.70	1246: 68798/71.340	1246: 21.3/10
1285	Protein disulfide isomerase	AT1G21750.2	PDIL1-1	Protein folding/refolding	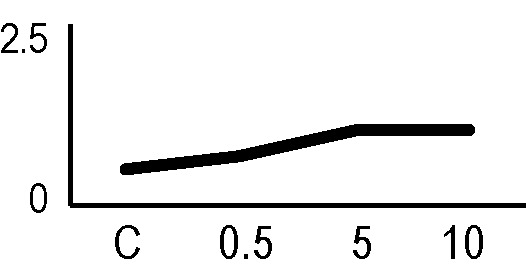	1285: 1.4; 2.1; 2.1	1285: 4.96/5.45	1285: 49.612/61.003	1285: 9.8/3
1285	ATPase V1 complex, subunit B	AT4G38510.5	VAB2	Amino acid metabolism			1285: 5.03/5.45	1285: 53.457/61.003	1285: 9.2/3
1531	Cysteine synthase	AT3G59760.3	OASC	Amino acid metabolism	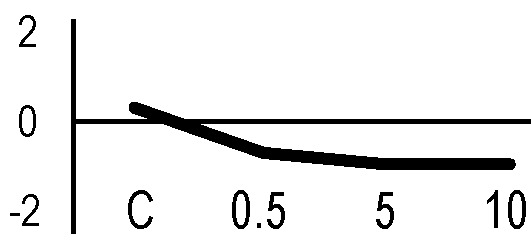	1531: 1; -1.7; -1.8	1531: 5.41/5.40	1531: 41255/51.067	1531: 22.5/6
1488 1539 1540	TCP-1/cpn60 chaperonin family protein	AT3G13470.1	Cpn60beta2	Protein folding/refolding	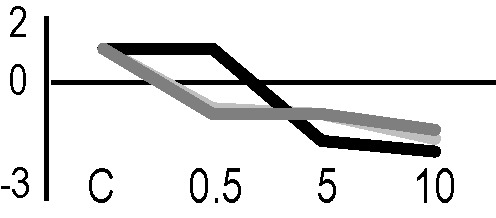	1488: -1.1; -1.2; -2.0 1539: 1.0; -2.0; -2.4 1540: -1.1; -1.2; -1.7	1539: 5.39/5.65 1540: 5.39/5.76	1488: 58.883/55.672 1539: 58.883/51.229 1540: 58.883/50.391	1488: 31.6/15 1539: 28.7/12 1540: 33.5/15
1539 1540	Aldehyde dehydrogenase	AT1G74920.1	ALDH10A8	Oxidation/reduction			1539: 5.33/5.64 1540: 5.33/5.76	1539: 56.545/51.229 1540: 56.545/50.391	1539: 22.8/9 1540: 29.3/13
1583	Monodehydro ascorbate reductase	AT3G52880.1	MDAR1	Oxidation/reduction	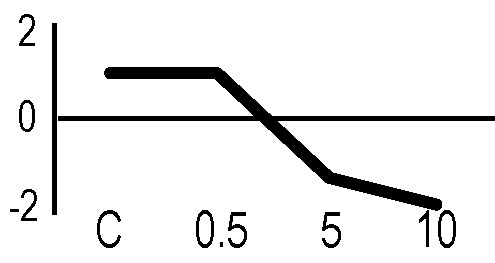	1583: 1.0; -1.2; -1.8	1583: 5.77/5.95	47.106/46.780	1583: 16.4/5
1631 1708 1826	Enolase	AT2G36530.1	LOS2	Carbon metabolism	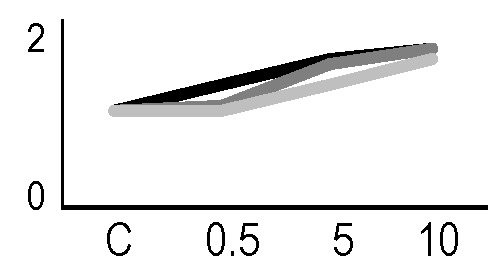	1631: 1.3; 1.6; 1.7 1708: 1.1; 1.5; 1.7 1826: 1.0; 1.3; 1.6	1631: 5.68/5.81 1708: 5.68/5.85 1826: 5.68/5.80	1631: 48.054/45.440 1708: 48.054/44.112 1826: 48.054/43.787	1631: 24.8/6 1708: 30.9/9 1826: 31.1/10
1631 1708 1826	Insulinase	AT1G51980.1	AT1G51980	Protein metabolism			1631: 6.05/5.81 1708: 6.05/5.85 1826: 6.05/5.80	1631: 54.869/45.440 1708: 54.869/44.112 1826: 54.869/43.787	1631: 17.7/7 1708: 29.8/11 1826: 24.6/9
1813	Glutathione-disulfide reductase	AT3G24170.3	At3g24170	Oxidation/reduction	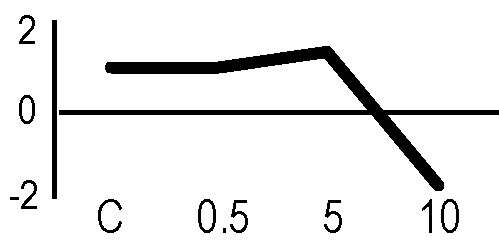	1813: 1.0; -1.3; -1.7	1813: 6.14/6.21	1813: 49.921/43.655	1813: 22.7/7
1987	S-adenosylmethionine synthase/	AT4G01850.2	SAM2	Amino acid metabolism	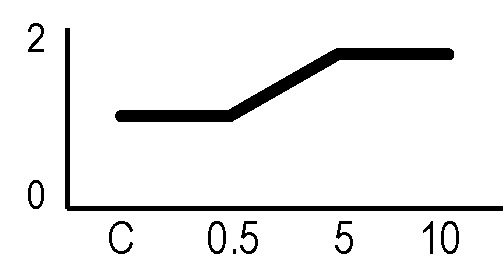	1987: 1.0; 1.7; 1.7	1987: 5.41/5.63	1987: 41.959/40.098	1987: 24.9/8
1987	Eukaryotic translation initiation factor	AT3G13920.1	EIF4A1	Protein metabolism			1987: 5.54/5.63	1987: 47.143/40.098	1987: 23.2/7
2084	Isocitrate dehydrogenase, cytosolic	AT1G65930.1	cICDH	Carbon metabolism	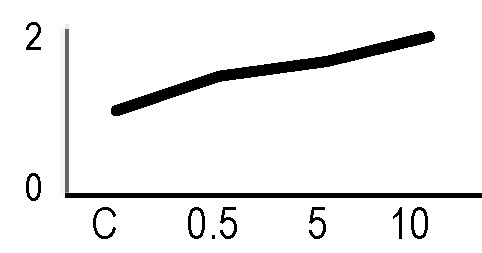	2084: 1.4; 1.6; 1.9	2084: 6.35/6.18	2084: 47.001/39.090	2084: 16.6/6
2133	Elicitor-activated gene 3-2	AT4G37990.1	ELI3-2	Response to stimuli/stress	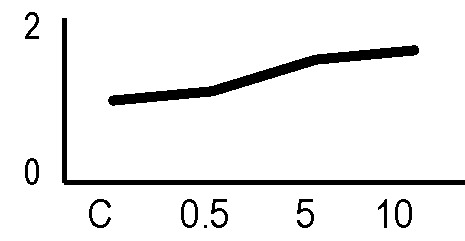	2133: 1.1; 1.5; 1.6	2133: 5.89/6.15	2133: 39.208/38.781	2133: 37.9/8
2133	Elongation factor Tu	AT4G02930.1	AT4G02930	Protein synthesis			2133: 6.54/6.15	2133: 49.257/38.781	2133: 42.4/12
2172	Glutamate dehydrogenase	AT5G18170.1	GDH1	Amino acid metabolism	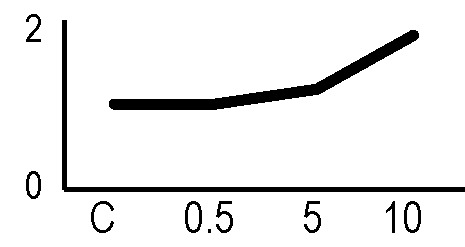	2172: 1.0; 1.2; 1.8	2172: 6.20/6.35	2172: 44.878/36.055	2172: 19.2/5
2536	Malate dehydrogenase	AT3G47520.1	MDH	Oxidation/reduction	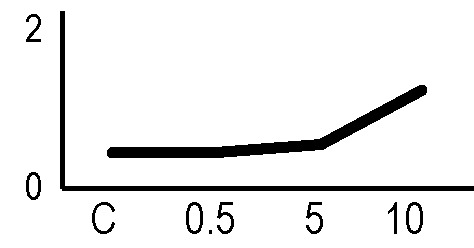	2536: 1.0; 1.2; 2.8	2536: 8.34/5.66	2536: 43.563/30.478	2536: 28.4/7
2761	Proteasome subunit alpha	AT5G42790.1	PAF1	Protein metabolism	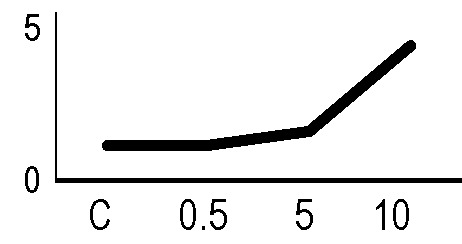	2761: 1.0; 1.5; 4.0	2761: 5.67/5.47	2761: 32.684/28.591	2761: 22.3/5
3154	Ascorbate peroxidase 2, cytosolic	AT1G07890.8	APX1	Oxidation/reduction	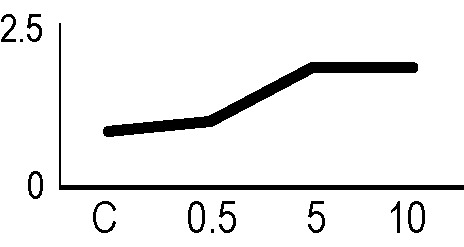	3154: 1.2; 2.2; 2.2	3154: 6.00/6.10	3154: 27.532/22.799	3154: 23.2/5
3394	Aconitase/3-isopropylmalate dehydrogenase	AT2G43090.1	AT2G43090	Oxidation/reduction	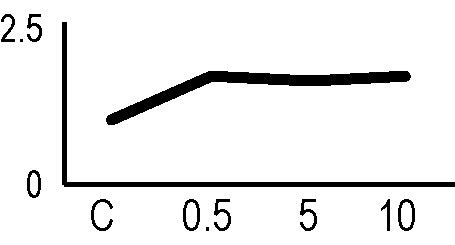	3394: 1.7; 1.6; 1.7	3394: 6.52/6.50	3394: 27355/19.321	3394: 25.8/4

^(1)^Relative protein abundance represented as graphic display of the Average Ratio values obtained from the statistical analysis and calculated as the ratio between the representativeness values of the single spot in the samples exposed to 0.5, 5, or 10 Gy and those obtained in the unexposed samples. ^(2)^Experimental (Exp) and theoretical (Theor) isoelectric point (pI) values. ^(3)^Experimental (Exp) and theoretical (Theor) molecular weight (MW). ^(4)^Protein sequence coverage (%) and number of unique peptides identified for each spot. Identification details (including Mascot score) are reported in **Supplementary Table S3**, while results from sequence alignment with respect to Arabidopsis thaliana counterparts are reported in **Supplementary Table S4** .

**Figure 4:**
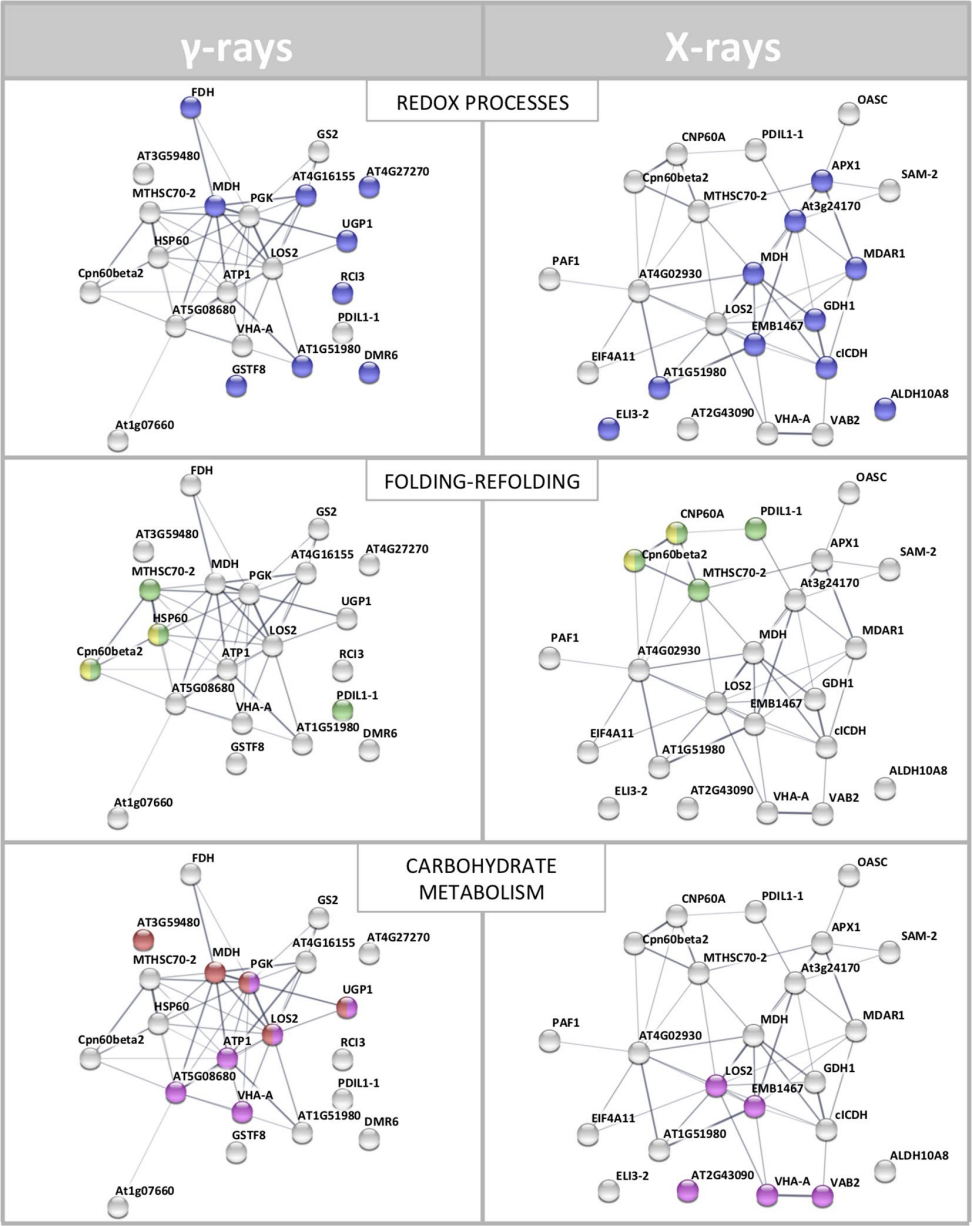
STRING meaningful functional enrichments, comparing the network of the proteomic response to γ-radiation and the network of the proteomic response to X-rays. Proteins involved in redox processes (blue), folding–refolding (green–yellow, respectively), and carbohydrate metabolic processes–carbohydrate derivative metabolic processes (red–fuchsia pink, respectively) are illustrated. Line thickness indicates the strength of data supporting the confidence of network edges.

To interpret the biological significance of the observed proteomic perturbations, functional interactions between above-mentioned DRPs were further investigated. PPI networks identified by STRING analysis showed highly significant functional associations (PPI enrichment *p*-value < 1.0^-15^, for both γ-ray and X-ray irradiation response analyses). The ontological analysis allowed to highlight a prevalence of proteins involved in stress response (redox reactions, protein folding/refolding and adaptive adjustment of carbohydrate, amino acid, nucleotide, and protein metabolism) in the data set from irradiation with both γ-rays and X-rays (**Figure 4**).

#### Proteins Involved in Redox Reactions

It is well known that ionizing radiations have a high penetration power and transmit their energy to the matter they pass through, causing ionization and excitation. When a biological tissue is exposed, damage is produced to macromolecules (i.e., proteins, nucleic acids, and lipids) and to cellular structures (i.e., membranes, chromosomes, and organelles) (De Micco et al., 2011). The effects vary according to many factors related to both the characteristics of the radiation and the specific biological response. Resulting phenomena are due not only to the direct passage of radiation but also as indirect consequence of the production of reactive oxygen species (ROS), such as superoxide radicals (O_2_^•–^), hydroxyl radicals (HO^•^) and H_2_O_2_, as well reductive species, such as solvated electrons (e_aq_^–^) and hydrogen atoms (H^•^). The latter species have been less investigated than the former ones due to the aerobic conditions most organisms generally experience (De Micco et al., 2011; Chatgilialoglu et al., 2011). When considering ROS, it is important to underline that they are normal products of plant cellular metabolism, also acting as second messengers in a variety of cellular processes regulating physiological response to environmental changes. However, when an excessive endogenous/exogenous production of ROS occurs, such in the case of stressful environmental conditions or abnormal exposition to radiations, the destructive potential of these molecules is exacerbated. In the latter contexts, the balance between ROS accumulation and their scavenging (due to antioxidant molecules) is altered, and the cell functionality is compromised (Mittler et al., 2011); this may cause an oxidative damage and ultimately cell death. In fact, ROS have been demonstrated rapidly reacting with and damaging almost all structural and functional molecules (proteins, nucleic acids, and lipids) (Chatgilialoglu et al., 2011; Gudkov et al., 2019). On the other hand, reductive stresses may also occur under specific environmental conditions, such as exposition to ionizing radiations in anaerobic conditions—i.e., the extraterrestrial scene, also contributing to alter cellular redox homeostasis (Ferreri et al., 2008; Salzano et al., 2011).

This proteomic study allowed to evaluate in detail the acclimation to the above-mentioned radiation stresses, giving a comprehensive indication of the protein representation modulation under these experimental conditions. Most of the observed protein variations were referable to components involved in the response to redox stress. In particular, our results revealed the activation of different redox balance control mechanisms in HRCs exposed to irradiation with γ-rays and X-rays.

The variation of some of the identified DRPs was associated with the direct effect of the radiation on cellular mechanisms directly promoting a quenching of the generated radical species. This is the case of ascorbate peroxidase (APX1), which was over-represented following exposure to X-rays. Scavenger function of APX1 was already studied in response to γ radiations, showing a significant activation only at high doses (58.8 Gy) (Vanhoudt et al., 2014). Under physiological conditions, APX1 activity is in equilibrium with that of monodehydroascorbate reductase (MDAR1), which converts back monodehydroascorbate to ascorbate. Sudan et al. (2015) demonstrated that, under mild stress conditions, including ultraviolet radiation, gene expression for peroxisomal MDAR1 and its catalytic activity are directly related, resulting in an increase in the enzyme activity. However, this correspondence tends to be lost at higher magnitudes of the imposed stress, with a concomitant reduction in MDAR1 activity. In HRCs, we observed a down-representation of MDAR1 as a consequence of X-ray exposure at 5 and 10 Gy. This observation, in apparent contradiction with the activation of APX1, may be explained by the consideration that the molecular oxidations induced by the radiation are inevitably associated with corresponding reductions (Reisz et al., 2014). On the other hand, the existence of a condition in which reductive stress can also occur at specific plant tissue sites after irradiation cannot be excluded at present. Even reduced species must be reported in equilibrium conditions to recover the redox state necessary for the cellular functionality, likely through the reduction of antioxidant activity. This hypothetical mechanism could explain also our results concerning glutathione-mediated quenching, which is closely correlated with ascorbate in detoxifying different cellular compartments. In fact, the reduced representation profiles we observed at 10 Gy X-rays for cytoplasmic glutathione-disulfide reductase (AT3G24170) could be analogously framed in the fine adjustment of the glutathione-ascorbate cycle at high radiation doses for the redox balance recovery (Nianiou-Obeidat et al., 2017). This evidence finds support in other works showing that glutathione-disulfide reductase post-stress modulation allows the glutathione pool to be regulated, thus determining the response to stress (e.g., in *Chlamydomonas* spp. exposed to intense light) (Lin et al., 2018). Similarly, we observed the down-representation of chloroplastic glutathione transferase (GSTF8) in HRCs exposed to γ-rays. This finding has already been reported in other works, and it was explained as a consequence of mechanisms of abiotic stress acclimation aimed at maintaining the pool and redox status of glutathione during detoxification processes (Csiszár et al., 2014).

Other enzymes involved in the stabilization of redox equilibrium were also down-represented after radiation exposure. This is the case of quinone reductase (AT4G27270), which catalyzes the transfer of electrons from NADH and NADPH by reducing quinone to the hydroquinone state. Similarly a cold inducible peroxidase (RCI3) was down-represented, in line with results obtained for *Arabidopsis* exposed to light (Llorente et al., 2002), suggesting a role of this redox enzyme also in acclimation response to radiation.

The oxidation–reduction imbalance caused by exposure to radiative stress affects the functionality of cellular organelles, in particular mitochondria and chloroplasts that must perform metabolic compensations to maintain their role vital for the cell. In particular, mitochondria are the site of respiratory processes, which include oxidation–reduction reactions and contribute to the formation of ROS through the direct reduction of oxygen to O_2_^•−^ in the flavoprotein region of NADH dehydrogenase of the respiratory chain (Sharma et al., 2012). In tomato HRCs, we verified the inhibition of NADH dehydrogenase (EMB1467) synthesis following exposure to X-rays, accomplished to contain the accumulation of ROS. Another enzyme identified as DRP and involved in cell respiration was formate dehydrogenase (FDH). FDH is a widespread enzyme abundant in non-green tissues and scarce in photosynthetic tissues. Under stress, FDH accumulates in leaf mitochondria, which acquire the ability to use formate as a respiratory substrate, through its oxidation in CO_2_. The lower representativeness of FDH following treatment with γ radiation may be referred once more to a fine metabolic modulation of the post-stress redox state (McNeilly et al., 2018).

Chloroplasts are extremely sensitive to ionizing radiations compared to other organelles (Wi et al., 2007). NAD-dependent malate dehydrogenases (MDHs) are oxidoreductases represented by various isoforms involved in different metabolic pathways. After exposure to both γ- and X-rays, we observed the over-representation of chloroplastic NADP-dependent MDH, which controls redox homeostasis between organelle compartments. Its activity is strictly redox regulated and influenced by light (Carr et al., 1999). Moreover, Du et al. (2011) demonstrated, through a proteomic analysis, a moderate increase in the expression of MDH in rice leaves exposed to UV-B and UV-A. In the same way, after γ irradiation, we observed the induction of dihydrolipoyl dehydrogenase (AT4G16155), which is an integral component of multienzyme systems and is involved in various processes of regulation of cell oxidative state (Timm et al., 2015). An opposite trend resulted after X-ray exposure for the chloroplastic betaine aldehyde dehydrogenases (ALDH10A8), belonging to a family of NAD(P)-dependent enzymes. ALDH10A8 can oxidize metabolism-derived aminoaldehydes, produced under stress conditions, to their corresponding amino acids; its increased synthesis is associated with cell detoxifying function (Missihoun et al., 2011). Although in contrast with what has been reported for other stresses, our results on this enzyme may be explained by a general down-regulation of oxidation reactions to compensate for a general oxidized cellular environment caused by radiations. Finally, the exposure to X-rays resulted in the over-representation of isopropylmalate dehydrogenase (AT2G43090). Although the role of this enzyme in the stress by abiotic factors is not clear at present, a mechanism involving it was already proposed, in which this protein was suggested to directly regulate glucosinolate metabolism associated with biotic stress response through thiol-based redox regulation (He et al., 2009).

#### Proteins Involved in Folding and Refolding

Interestingly, our analysis also found representation changes in a number of components involved in protein folding processes. This observation is in agreement with the hypothesis on the activation of cellular mechanisms to buffer radiation damages, which likely require the synthesis of novel proteins. In fact, stress survival is linked to the cell’s ability to compensate for dysfunction, through molecular mechanisms involving the maintenance of proteins in their functional conformation and the prevention of non-native protein aggregation. An important role in this context is played by heat shock proteins, chaperones responsible for the folding, assembly, translocation, and degradation of proteins during normal conditions or after stress (Wang et al., 2004). In our study, we verified the modulation of different heat shock proteins (belonging to the TCP-1/cpn60 and HSP70 families) as molecular and dynamic tools aimed at restoring polypeptide function homeostasis. The involvement of heat shock proteins in the response to radiation was already documented only for UV rays, identifying regulatory relationships between the responses of plants to heat stress and UV damage (Swindell et al., 2007). Heat shock proteins are also active in protein refolding, so their role in repairing protein structures directly damaged by radiation is conceivable. One additional example of this function is represented by protein-disulfide isomerase (PDIL1-1), which was also over-represented in HRCs exposed to γ- and X-rays. It assists protein folding, facilitating intramolecular rearrangements in which disulfide bonds are broken and formed. PDIL1-1 activation in stress conditions is effective both in the synthesis of novel proteins to counteract functional imbalances and in preventing/repairing harmful effects of ROS through a maintenance of the protein/cellular redox state (Zhang et al., 2018).

#### Proteins Involved in Carbon and Amino Acid Metabolism

Other DRPs resulting from this study are more generally involved in mechanisms of metabolic adaptation to stress conditions. The sophisticated mechanisms put in place by plants to cope with stresses involve changes in sugar homeostasis, which allow these organisms to respond quickly to environmental alterations. Coordinated processes allow to modulate the metabolism of carbohydrates that perform different functions under stress conditions, such as stabilizing membranes and proteins. Furthermore, the modification (phosphorylation) of sugars (glucose and fructose) transforms it into highly functional molecules, directing them toward the oxidative pentose phosphate pathway. Moreover, through the biosynthesis of sugar alcohols, i.e., sorbitol or mannitol, very efficient ROS scavengers are produced (Pommerrenig et al., 2018).

In this context, we observed the over-representation of enolase (LOS2) following exposure to both γ and X radiations. LOS2 is a multifunctional enzyme involved in carbohydrate metabolism, whose role in the response to various abiotic stresses is well documented (Kosová et al., 2018). Casati and Walbot (2003) demonstrated that UV-B radiation increases the level of transcripts for enolase in maize genotypes to provide additional energy to counteract stress. Interestingly, a function in post-transcriptional control could also be ascribed to enolase. In fact, it takes a part in a multienzyme complex called RNA degradosome, which plays an important role in RNA processing, thus allowing to regulate the stress response (Weng et al., 2016). Fructokinases are also involved in the mobilization of sugars and are represented in plant by a family of enzymes playing important roles in regulating the amount of carbohydrates metabolized in different tissues under challenge conditions (Granot et al., 2014). The increase in fructokinase-2 (AT3G59480) representation levels obtained after γ-rays exposure should likely refer to the need of the plant to metabolize hexose sugars through phosphorylation, thus providing energy necessary to sustain the corresponding stress response.

Sugars also play an important role in the metabolic signaling system. In particular, UDP-glucose has been proposed as a potential intracellular mediator of ROS signaling and programmed cell death. Its synthesis is due to enzymes like UDP-glucose pyrophosphorylase (UGP1), whose expression is regulated under stress conditions (Janse van Rensburg and Van den Ende, 2018). In our analysis, HRCs reacted to γ-rays overproducing UGP1. Another DRP that could be involved in the redox signaling is cytosolic isocitrate dehydrogenase (CICDH), which was over-represented after radiation. CICDH promotes homeostasis in response to oxidative stress and its induction may allow the recycling of NADPH as a mechanism against cellular oxidative damage (Valderrama et al., 2006).

Amino acid metabolism was also activated in HRCs exposed to ionizing radiation. The association between the amino acid synthesis and plant responses to abiotic stress has been already documented (Batista-Silva et al., 2019). Indeed, amino acids function as either nitrogen stores or precursors for secondary metabolites. We observed an increase in glutamine synthetase (GS2) representation, in agreement with the results of Gicquel et al. (2011), which were obtained through a proteomic study on *Arabidopsis* exposed to X-rays. GS2 is a photorespiratory enzyme and acts as a regulator of nitrogen metabolism, assimilating ammonium into amino acids and regulating between the nitrogen and the carbon cycles *via* maintaining glutamine–glutamate pool in the chloroplast. We also found a variation in S-adenosylmethionine synthase (SAM2) representation. This enzyme catalyzes the formation (from methionine and ATP) of S-adenosylmethionine, a coenzyme involved in methylation processes regulating protein function. Its expression correlates with exposure to stress (Ma et al., 2017) and is therefore in agreement with our results obtained after X-radiation. Among the mitochondrial enzymes, we observed the differential representation of the glutamate dehydrogenase (GDH1), which converts glutamate to α-ketoglutarate and *vice versa*, depending on the environment and the applied stress. In accordance with our results, Skopelitis et al. (2006) demonstrated that ROS signal induces GDH expression, acting as anti-stress enzymes in ammonia detoxification. An opposite trend was observed for O-acetylserine (thiol) lyase (OASC) that catalyzes the transfer of the –SH group to acetylserine, which further split into acetate and cysteine. Together with serine acetyltransferase, OASC forms the functional complex cysteine synthase, whose functionality is modulated in the plant cell depending on environmental conditions (Hell and Wirtz, 2011). In fact, cysteine is essential not only for protein synthesis but also for the formation of the antioxidant compound glutathione (Gallardo et al., 2014). The reduced expression of OASC, which we observed following exposure to X-rays, may be framed in balancing mechanisms of these regulatory pathways related to sulfur metabolism.

More generally, the activation of different metabolic pathways involves adenosine triphosphate (ATP), such as universally important coenzyme and enzyme regulator (De Col et al., 2017). As in many other response mechanisms, we also verified the activation of ATP metabolic processes due to the exposure to ionizing radiations. In particular, in response to radiation stress, we found alterations of the representation of various ATP synthase isoforms (ATP1; AT5G08680; VAB2; VHA-A) located in different cell compartments, i.e., cytosol, mitochondria, chloroplast, and vacuole. It is well known that the expression modulation of members of this protein family promotes energy-demanding processes, although specific references to plant response to ionizing radiation are not present in the scientific literature. Moreover, we found the overrepresentation after γ radiation of phosphoglycerate kinase (PGK), an enzyme involved in ATP biosynthesis through the high-energy phosphoryl transfer of the acyl phosphate of 1,3-bisphosphoglycerate to ADP.

#### Proteins Involved in Protein Metabolism

Proteomic analysis of irradiated tomato HRCs also revealed the variation in representation of proteins somehow involved in transcription and translation processes. Among these DRPs, we observed the overrepresentation after X-rays of the translation initiation factor (EIF4A1), which is associated with plant response to different abiotic stresses (Dutt et al., 2015). Similarly, the elongation factor Tu (AT4G02930) promotes the binding of aminoacyl-tRNA to the A-site of ribosomes during protein biosynthesis. Its observed over-representation correlated with an enhanced tolerance to abiotic stresses (Fu et al., 2012). Finally, the over-representation of a chaperone involved in the assembly of nucleosomes, histone H4 (AT5G59970), was also detected after irradiation. The involvement of epigenetic regulators in oxidative stress has been widely reported; in particular, adverse conditions affect the degree of acetylation of histone H4, thus allowing the regulation of gene expression (Luo et al., 2017). Variation of histones expression levels in relation to stress is not documented, but it is known that X-rays induce structural damage in the H4 histone of HeLa cells, with consequences on transcription regulation (Izumi et al., 2017). Therefore, a possibility exists that such damage may be reflected in a corresponding increased synthesis of this protein to restore correct transcriptional activity, which is indispensable for dealing with damage to macromolecules caused by radiation.

Another protein differentially represented after exposure to ionizing radiation is insulinase (AT1G51980), which is homologous to the superfamily of mammalian insulin-degrading enzymes. Although its role in plant is not yet clear, the remarkable structural and functional conservation of the proteasome between plants, fungi, and animals suggests common mechanisms regulating proteasome activity in response to environmental modifications (Fu et al., 1999). The involvement of the proteasome in the response of the HRCs to ionizing radiations was also highlighted by the over-representation of proteasome subunit alpha (PAF1). Actually, proteasome is involved in cellular degradation processes of oxidized proteins, thus increasing oxidative stress tolerance (Kurepa et al., 2009). The effect of ionizing radiation on the proteasome was studied on cultured human cells, revealing a functional dose-dependent response (Pajonk and McBride, 2001).

### Validation of the 2D-DIGE Results by Immunoblot Analysis

**Figure 5:**
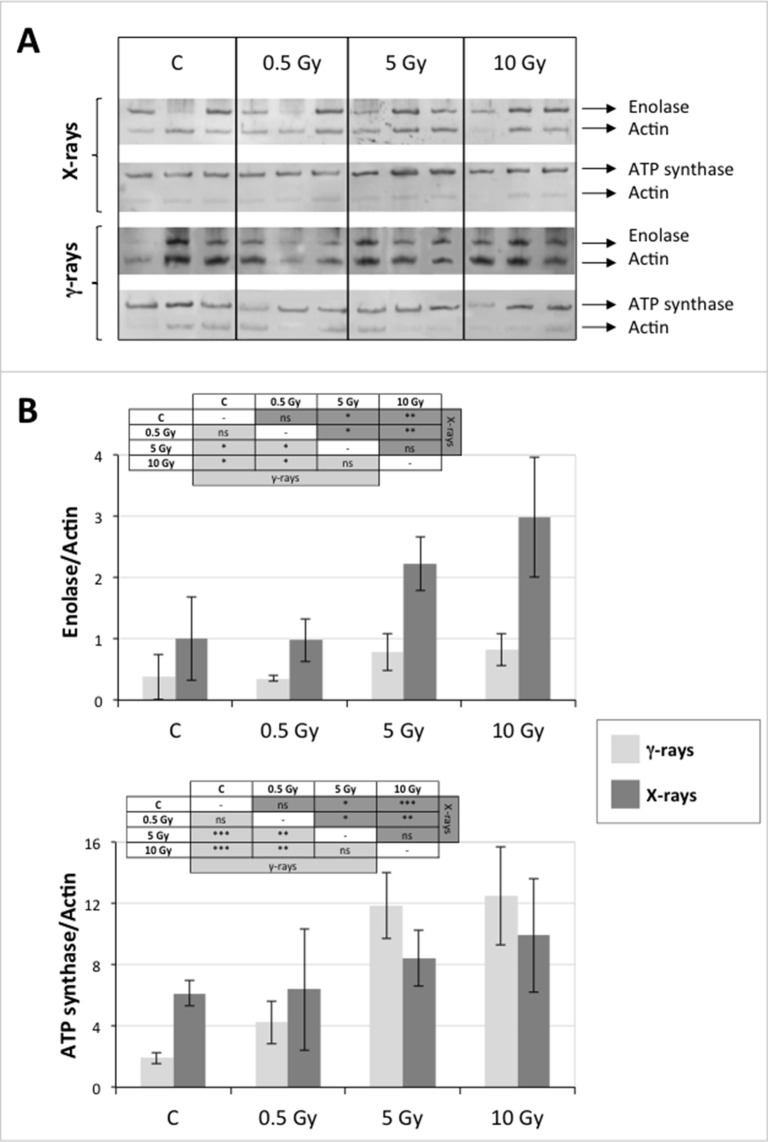
Immunoblotting analysis performed to validate 2D-DIGE results. **(A)** Specific polyclonal antibodies were used to detect the expression of enolase and ATP synthase in HRCs, independently exposed to X- and γ-radiation. Actin expression was used to normalize protein quantity. **(B)** Immunoblot signals were quantified by densitometric analysis. Data are presented as means values obtained from three biological replicates ± standard deviation. Tables show the results of one-way ANOVA followed by Fisher’s least significant difference (LSD) test, based on three replication sample dimension. Statistical significance is indicated with asterisks: ∗ = p < 0.05; ∗∗ = p < 0.01; ∗∗∗ = p < 0.001. ns: no statistically significant differences.

In order to validate the results obtained from the 2D-DIGE analysis, two proteins differentially represented after irradiation with either γ-rays or X-rays were selected, namely enolase (LOS2) and chloroplastic ATP synthase subunit B (AtpB). Both enzymes are involved in plant carbon/energy metabolism, playing an active role in the production of energy necessary to support the acclimation processes. Densitometric analysis of the bands obtained by immunoblotting (**Figure 5A**) was supported by statistical processing to measure the dose-dependent expression levels of the two proteins both in samples exposed to γ-rays and in those exposed to X-rays (**Figure 5B** and **Supplementary Table S5**). The results confirmed the expression trends obtained by the proteomic analysis for both DRPs (**Figure 5**). In detail, protein representation levels obtained at 0.5 Gy were comparable to those of the control HRCs not exposed to radiation. An increase in the concentration of LOS2 and AtpB in the soluble protein was instead evident at 5 and 10 Gy. The good correlation between 2D-DIGE and immunoblotting contributed to making the obtained dataset of differentially represented proteins technically solid and biologically significant.

### Effects of Ionizing Radiation on Hairy Roots Growth

**Figure 6:**
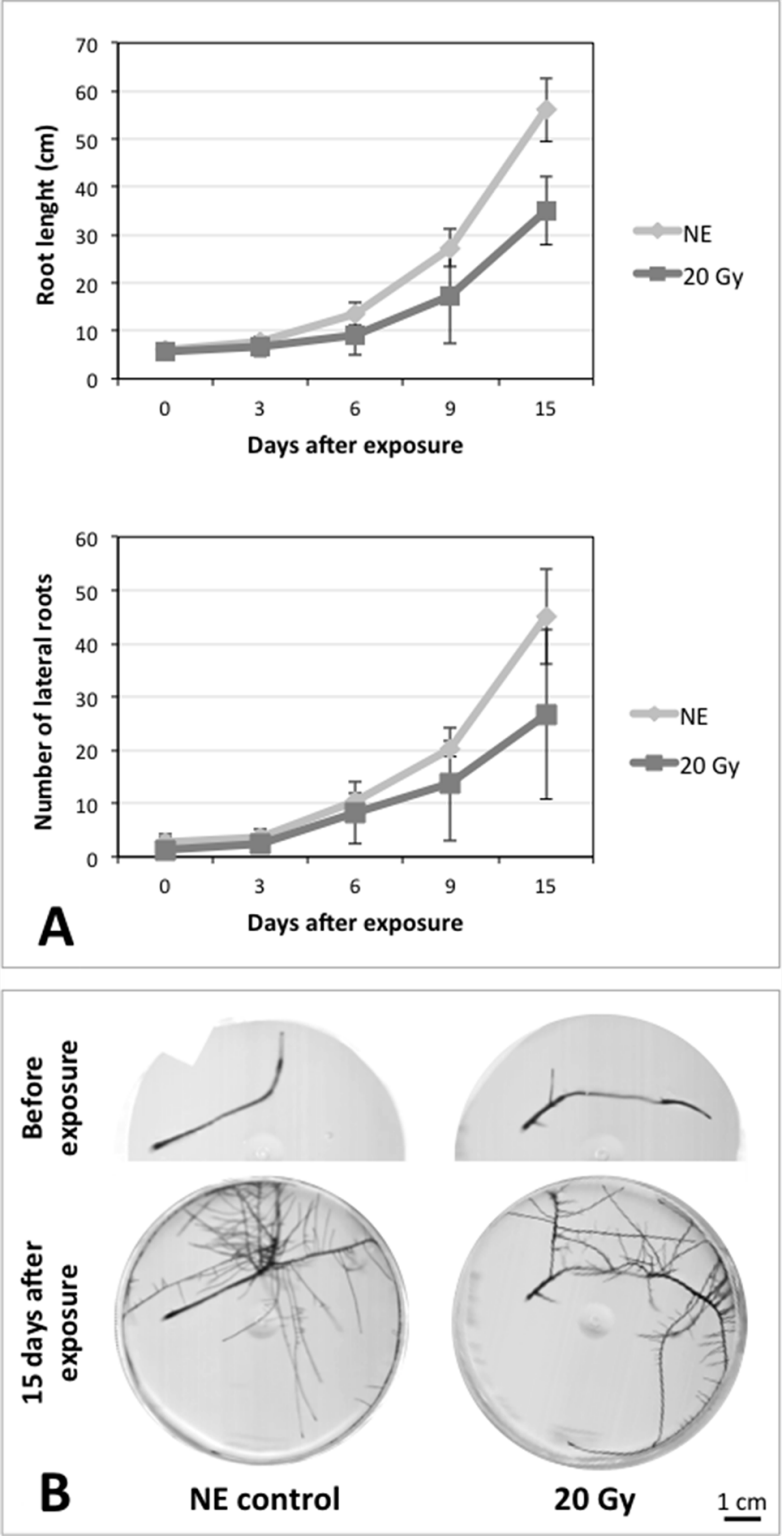
Results of morphometric analysis of HRCs exposed to 20 Gy γ-radiation. **(A)** Hairy root growth was measured at 3, 6, 9, and 15 days after exposure. The total root length and the number of lateral roots of irradiated HRCs (indicated in the graphs as 20 Gy) were compared with not exposed control (indicated in the graphs as NE). Data are presented as mean values obtained from three biological replicates ± standard deviation. **(B)** Examples of HRCs used for biometrical analysis.

After the analysis of proteomic response to ionizing radiations, we investigated if the alterations in protein expression levels, and the related consequences on the metabolism, could have effects on the root culture growth or even compromise the survival of these plant tissues. Accordingly, we decided to perform a morphometric analysis of HRCs exposed to γ-rays, following post-stress culture growth in optimal environmental conditions. For this experiment, a higher dose of radiation (20 Gy) was chosen, in order to make more evident any deleterious effects of metabolic processes triggered by radiative stress. The results showed that radiation caused a general reduction in root development with respect to unexposed control roots, which was measured in terms of total root length and number of lateral roots characterizing the hairy roots morphology (**Figure 6**). This alteration was noticeable only one week after the exposure. Despite this reduced growth, the HRCs did not undergo necrotic phenomena and showed no evident signs of tissue alteration.

## Conclusions

This study shows that the proposed plant “ideotype”, represented by tomato HRCs accumulating antioxidant pigments, tolerated ionizing radiations, at doses corresponding to the total radiation absorbed during a mission in the ISS (i.e., approximately 0.5 Gy in 6 months), without showing any alteration of protein representation profiles. At 10- and 20-fold higher radiation doses, a series of metabolic processes were activated, which are associated with the response to stress, and, in particular, with redox stress. Substantially similar responses were observed after plant exposure to irradiation with γ- and X-rays. The biological significance of these protein representation changes is likely attributable to a metabolic adaptation aimed at acclimation to extreme conditions. This hypothesis was supported by the absence of dramatic effects on the morphological development of the roots, even after several days after exposure. These experimental evidences well correlate with the results previously obtained on the tolerance of the same hairy roots system to high-intensity static magnetic fields (Villani et al., 2017). Therefore, this study supports the use of this plant system as a biofactory for the production of ready-to-use bioactive molecules directly in orbiting or planetary stations.

## Data Availability Statement

The datasets generated for this study can be found in the PXD014748.

## Author Contributions

AD and MV contributed to the conception and design of the experiments, performed proteomics experiments, carried out the data analysis, and wrote the manuscript. AMS and AS performed mass spectrometry analysis, interpretation of the data, and critical draft correction. SM prepared and characterized tomato hairy roots. MP and VC performed gamma irradiation of hairy roots. VDC performed X irradiation of hairy roots. LN set up and controlled the thermostatic chambers for hairy roots cultivation and performed immunoblotting statistical analysis. EB was the scientific director of the project and coordinated the activities of the research group. All authors approved the final version of the manuscript.

## Funding

This work was partially funded by the Italian Space Agency (ASI) (Grant No. 2014-007-R.0; Grant No. 2017-35-H.0).

## Conflict of Interest

The authors declare that the research was conducted in the absence of any commercial or financial relationships that could be construed as a potential conflict of interest.
