# Supplementary figures and images for: Effects of Simulated Space Radiations on the Tomato Root Proteome
Source: Front Plant Sci. 2019 Oct 24;10:1334. doi: 10.3389/fpls.2019.01334 (PMC6821793; doi:10.3389/fpls.2019.01334)

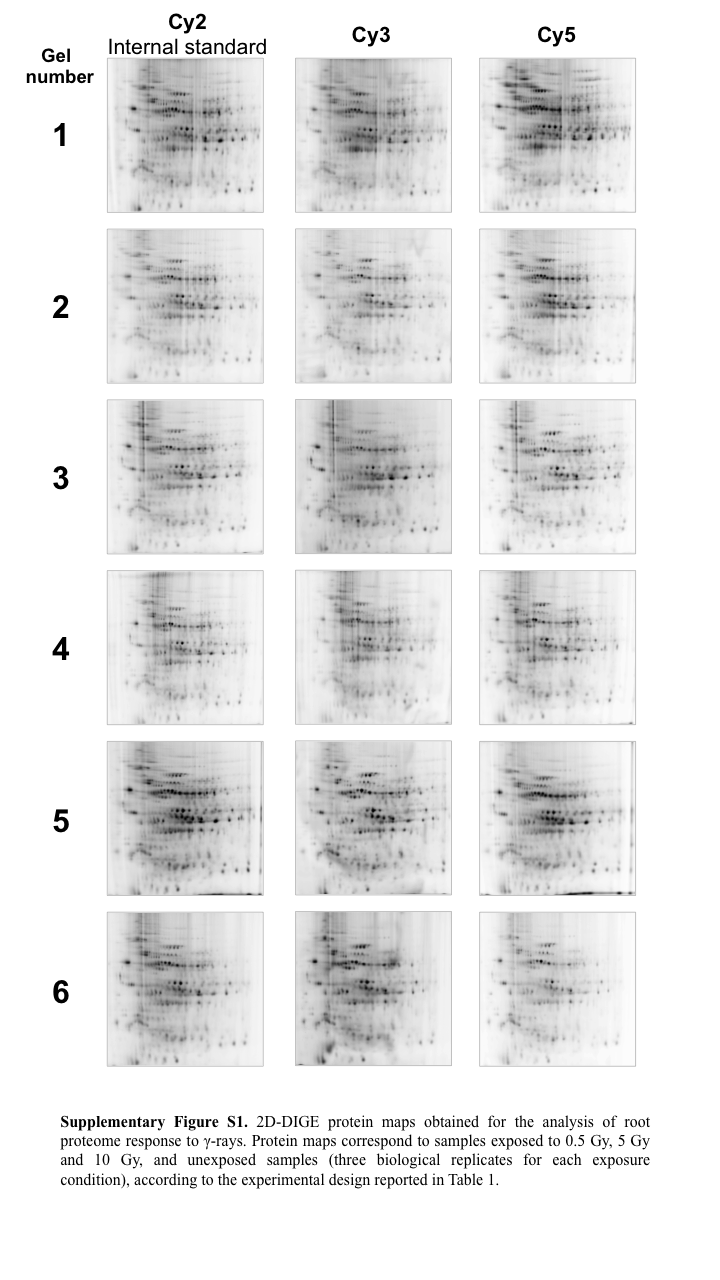

Supplement: Supplementary file 1 [file Image_1.tif]

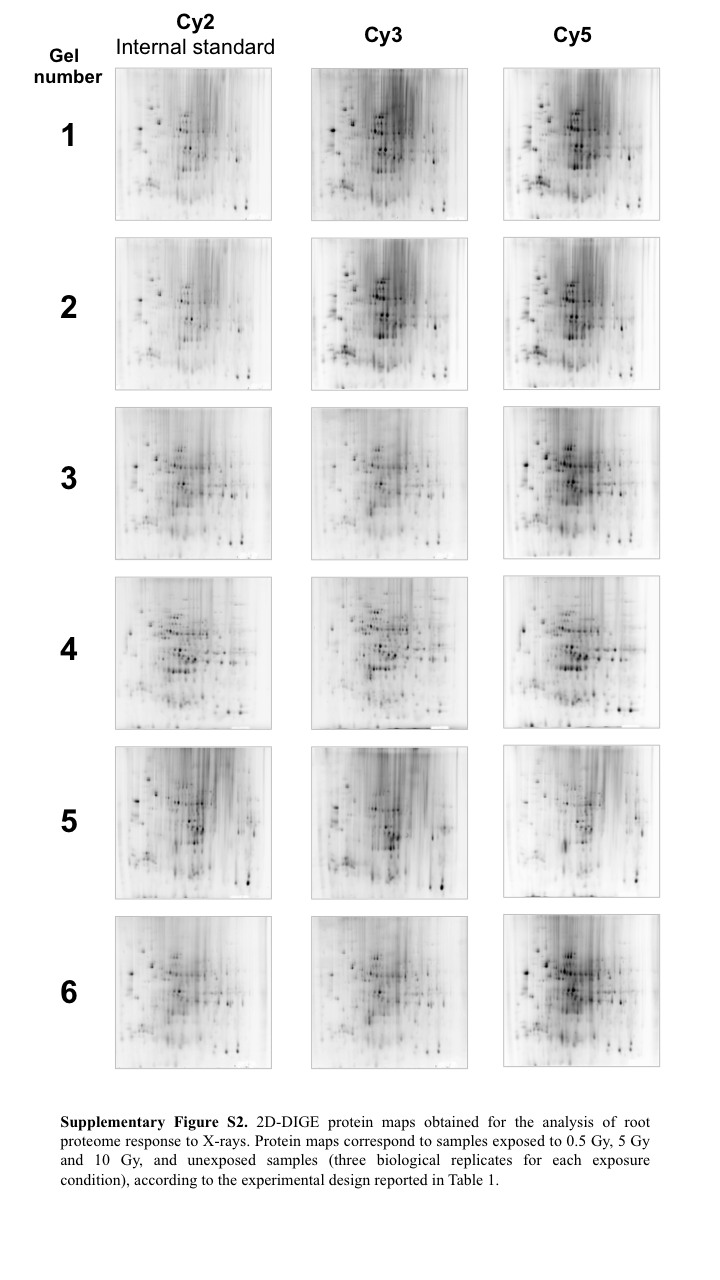

Supplement: Supplementary file 2 [file Image_2.tif]
